# Photoswitchable gating of non-equilibrium enzymatic feedback in chemically communicating polymersome nanoreactors

**DOI:** 10.1038/s41557-022-01062-4

**Published:** 2022-11-07

**Authors:** Omar Rifaie-Graham, Jonathan Yeow, Adrian Najer, Richard Wang, Rujie Sun, Kun Zhou, Tristan N. Dell, Christopher Adrianus, Chalaisorn Thanapongpibul, Mohamed Chami, Stephen Mann, Javier Read de Alaniz, Molly M. Stevens

**Affiliations:** 1 Department of Materials, Department of Bioengineering and Institute of Biomedical Engineering, Imperial College London, London, UK; 2 BioEM lab, Biozentrum, University of Basel, Basel, Switzerland; 3 Centre for Protolife Research and Centre for Organized Matter Chemistry, School of Chemistry, University of Bristol, Bristol, UK; 4 School of Materials Science and Engineering, Shanghai Jiao Tong University, Shanghai, China; 5 Max Planck-Bristol Centre for Minimal Biology, School of Chemistry, University of Bristol, Bristol, UK; 6 Department of Chemistry and Biochemistry, University of California, Santa Barbara, CA USA

**Keywords:** Synthetic biology, Polymer chemistry, Photocatalysis, Photocatalysis

## Abstract

The circadian rhythm generates out-of-equilibrium metabolite oscillations that are controlled by feedback loops under light/dark cycles. Here we describe a non-equilibrium nanosystem comprising a binary population of enzyme-containing polymersomes capable of light-gated chemical communication, controllable feedback and coupling to macroscopic oscillations. The populations consist of esterase-containing polymersomes functionalized with photo-responsive donor–acceptor Stenhouse adducts (DASA) and light-insensitive semipermeable urease-loaded polymersomes. The DASA–polymersome membrane becomes permeable under green light, switching on esterase activity and decreasing the pH, which in turn initiates the production of alkali in the urease-containing population. A pH-sensitive pigment that absorbs green light when protonated provides a negative feedback loop for deactivating the DASA–polymersomes. Simultaneously, increased alkali production deprotonates the pigment, reactivating esterase activity by opening the membrane gate. We utilize light-mediated fluctuations of pH to perform non-equilibrium communication between the nanoreactors and use the feedback loops to induce work as chemomechanical swelling/deswelling oscillations in a crosslinked hydrogel. We envision possible applications in artificial organelles, protocells and soft robotics.

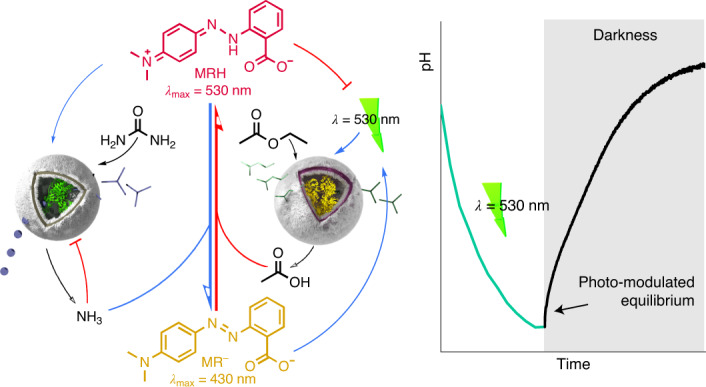

## Abstract (web summary)

The circadian rhythm generates out-of-equilibrium metabolite oscillations controlled by feedback loops under light/dark cycles. Now, it has been shown that these life-like properties can emerge from a non-equilibrium nanosystem comprising a binary population of enzyme-containing polymersomes capable of light-gated chemical communication, controllable feedback and coupling to macroscopic oscillations.

## Main

Most life-distinguishing features rely on complex molecular assemblies and biochemical reactions that are out of equilibrium^1^. This is possible in living systems owing to a higher level of continuous kinetic control and energy dissipation that is mediated by complex feedback loops. For example, a vast number of physiological functions in organisms are controlled by time-regulated enzymatic reactions. One prominent example is the circadian rhythm, which uses day and night cycles to regulate the alternation of metabolic activity. In these processes, the oscillation of metabolite concentrations is controlled by chemical hierarchical networks of independent oscillators that communicate and regulate each other to adapt to light intensity. Given that metabolites dissipate at a constant rate, the oscillation of physiological functions requires the accumulation of chemical signals through positive feedback loops and the inhibition of their production through negative feedback loops. This leads to the out-of-equilibrium state commonly referred to as homeostasis. The complex and dynamic interplay of these feedback loops in regulating metabolite levels is one of the fundamental hallmarks of living systems and can serve as inspiration for the synthesis of artificial out-of-equilibrium systems.

Polymersomes are self-assembled block copolymer vesicles that can mimic the compartmentalization of enzymes by cells and organelles, and can be tailored to be permanently semipermeable to small-molecule substrates or to switch semipermeability states in response to chemical and physical stimuli^2–5^. Such properties can be harnessed to generate enzyme-loaded polymersome nanoreactors that only perform catalysis when they are in an out-of-equilibrium state in response to stimuli^6–10^. One method that living systems have evolved to maintain these out-of-equilibrium processes is to control the transport of metabolites through chemically selective channels. In mimicking the activity of such stimuli-responsive channels within artificial systems, small-molecule photoswitches are especially attractive due to their ability to change geometry, polarity and absorption profiles both in solution and in the solid state^11^. This property allows for external spatial and temporal control over molecular systems by simply applying light in a non-invasive manner^12^. Examples of these systems include polymersomes functionalized with photoresponsive azobenzene or spiropyran moieties^13,14^. However, generally, these systems only respond to the ultraviolet (UV) or the blue region of the visible spectrum, and timescales for isomerization can vary greatly from minutes to days^15^, unlike donor–acceptor Stenhouse adduct (DASA) systems, which have all been reported to exhibit switching timescales of minutes^16–18^. DASAs are negative photoswitches that shift equilibrium between two isomers^19,20^. In the dark, the equilibrium is shifted to a colourful triene-enol, and under visible-light irradiation, a colourless cyclopentenone isomer that is more polar is formed. These features make DASAs highly attractive for robust implementations into different materials for widespread applications^21–29^. For example, coupling DASAs to the hydrophobic leaflet of polymersomes allows for an out-of-equilibrium state under light irradiation that permits the permeation of molecules across the now semipermeable membrane, recovering to the initial non-permeable state when light is withdrawn^7^.

**Fig. 1:**
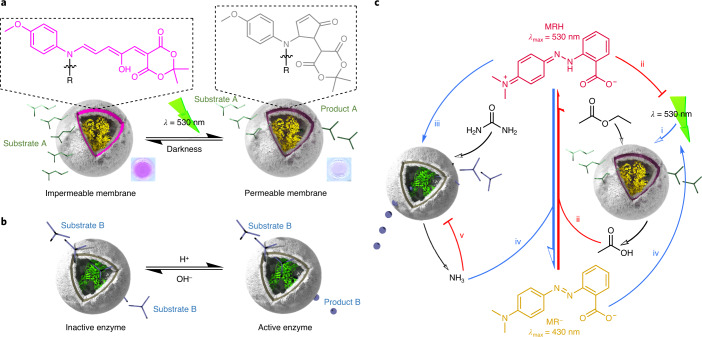
DASA photoswitch-driven enzymatic feedback loops between communicating polymersome nanoreactors. **a**, The catalytic activity of DASA–esterase nanoreactors is switched on by irradiation of green light and switched off by the withdrawal of light. The substrate permeates through the membrane when the colourful and non-polar triene-enol DASA isomer (shown in pink) is photoisomerized to the more polar and colourless cyclopentenone form (shown in grey). **b**, PISA–urease nanoreactors are permanently permeable to small molecules such as urea and ammonia. The enzyme is optimally active under acidic conditions and inactive under basic conditions. **c**, Negative and positive feedback loops generated between nanoreactors containing antagonistic enzymes. (i) DASA–esterase nanoreactors generate acetic acid by enzymatic hydrolysis of ethyl acetate in the presence of green light. (ii) The acid generates MRH from MR, which shifts its absorbance to the green region of the visible spectrum and masks the penetration of further green light, inhibiting its own synthesis. (iii) The acidification activates PISA–urease nanoreactors, which enzymatically hydrolyse urea into ammonia. (iv) The ammonia promotes the formation of MR^−^, allowing further penetration of green light and the production of acetic acid. (v) The accumulation of ammonia inhibits further hydrolysis from PISA–urease. Blue arrows represent promotion reactions; red arrows represent inhibition reactions.

Inspired by circadian rhythm processes, we designed feedback loops that work in tandem to facilitate modulation of out-of-equilibrium pH states powered by a DASA-functionalized polymersome nanoreactor containing an esterase enzyme (DASA–esterase; Fig. 1a). DASAs mimic photoreceptor-coupled transmembrane channels in living cells by controlling the permeation of substrates through a semipermeable membrane under light. Thus, DASA–polymersomes are ideally suited to modulate feedback loops externally triggered by light. When DASA–esterase was irradiated with green light, the polymersome membrane became semipermeable to facilitate access of the substrate (ethyl acetate) to the encapsulated enzyme for the generation of acetic acid. The first negative feedback loop within this system was produced by coupling this formation of acid with the pH-sensitive pigment methyl red (MR), which transitioned to a green-light-absorbing species as the pH was lowered. As acetic acid was catalytically formed under green light, the formation of the protonated pigment competed with the DASA–nanoreactor for the absorption of light until the catalysis was interrupted by deactivation of the photoswitching capacity of the DASA. The second level of regulation was introduced by an intrinsically semipermeable polymersome synthesized by polymerization-induced self-assembly (PISA)^30^, which encapsulated a urease enzyme (PISA–urease; Fig. 1b). Although inactive under basic conditions, acidic conditions drastically increased urease activity to transform its substrate—urea—into basic ammonia (Supplementary Fig. 1 and Supplementary Section 1)^31–35^. This formation of base drives the deprotonation of MR (MR^−^) back to its non-green-light-absorbing state (Fig. 1c). Therefore, the concentration of the green-absorbing protonated MR (MRH) could be modulated by irradiation or withdrawal of green light. Importantly, the control of MRH concentration was associated with light-controlled pH states. This further allowed the modulation of the swelling ratio of a pH-responsive hydrogel, which was immersed in the same medium as the nanoreactors, by transduction of green light into an out-of-equilibrium chemomechanical signal.

## Results and discussion

### Synthesis of antagonistic polymersome nanoreactors

**Fig. 2:**
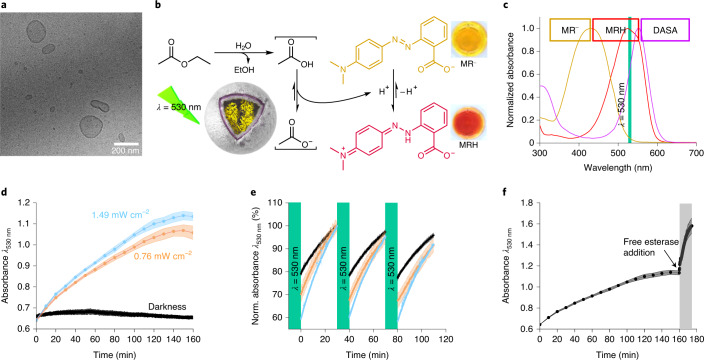
Green-light-mediated modulation of DASA–esterase catalytic activity and negative feedback loop generation. **a**, Cryo-TEM micrograph of DASA–esterase nanoreactors. **b**, Schematic representation of the biocatalytic hydrolysis of ethyl acetate to produce acetic acid by DASA–esterase nanoreactors. The production of acid accumulated MRH, generating a transition from yellow solutions in basic conditions to red in acidic conditions. **c**, UV–vis spectral scans of MR^−^ (yellow), MRH (red) and an organic DASA polymer solution (violet). **d**, UV–vis measurements of the DASA-esterase nanoreactor-mediated biocatalytic hydrolysis of ethyl acetate in the presence of MR by continuous irradiation of green light at 1.49 mW cm^−2^ (blue), 0.76 mW cm^−2^ (orange) and in darkness (black). The protonation of MR was monitored at 530 nm (*n* = 3 technical replicates, mean ± s.d.). **e**, UV–vis measurements of DASA polymersome photoswitching by alternating irradiation with green light and recovery of absorbance in the dark in the absence of MR. In each cycle, the dispersions were irradiated for 10 min and thermal recovery of the absorbance was monitored at 530 nm for 30 min. The samples were irradiated at light intensities of 0.76 mW cm^−2^ (black), 1.49 mW cm^−2^ (orange) and 2.12 mW cm^−2^ (blue) (*n* = 3 technical replicates, mean ± s.d.). **f**, Addition of free esterase after formation of plateau mediated by DASA–esterase nanoreactors irradiated with green light. The collection of data as in **d** at 1.49 mW cm^−2^ was repeated, with the addition of 60 pmol of free esterase after 160 min of green light irradiation (*n* = 3 technical replicates, mean ± s.d.). Source data

**Fig. 3:**
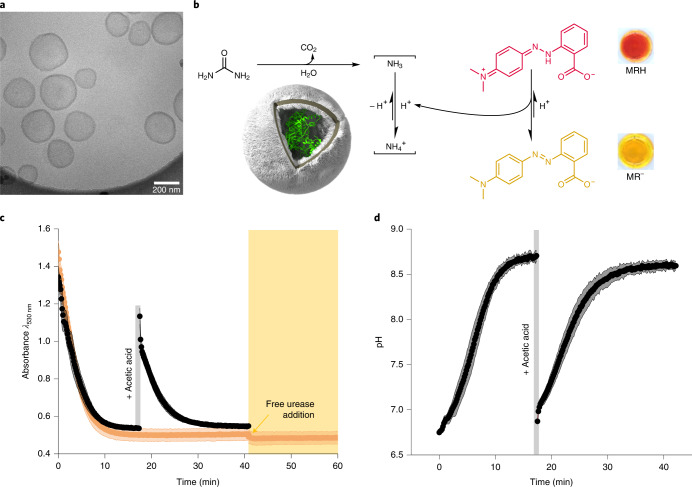
pH-mediated automodulation of PISA–urease catalytic activity. **a**, Cryo-TEM micrograph of PISA–urease nanoreactors. **b**, Schematic representation of the biocatalytic hydrolysis of urea to produce ammonia by PISA–urease nanoreactors. The production of base produces MR^−^, generating a transition from red solutions under acidic conditions to yellow under basic conditions. **c**, UV–vis measurements of PISA–urease-mediated biocatalytic hydrolysis of urea. The formation of ammonia was monitored by the production of MR^−^ at 530 nm. Orange: after 40 min, 0.17 nmol of free urease was added (*n* = 3 technical replicates, mean ± s.d.). Black: the formation of MR^−^ was monitored for 17 min, followed by addition of 24 nmol of acetic acid, then the production of MR^−^ was monitored again (*n* = 3 technical replicates, mean ± s.d.). **d**, pH monitoring of ammonia formation by PISA–urease nanoreactors calculated from UV–vis traces (black) in **c**. Source data

**Extended Data Fig. 1:**
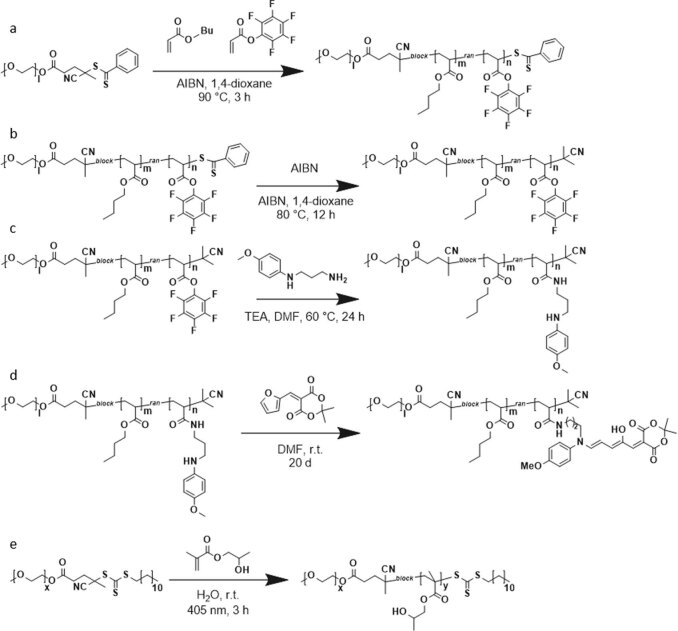
Synthesis of functional polymers. **a**. Chain extension of PEG macroCTA with PFPA and BA by RAFT polymerisation producing the activated ester diblock copolymer. **b**. Removal of the chain-transfer agent end-group from the activated ester diblock copolymer. **c**. Synthesis of the aromatic amine DASA precursor polymer by amidation with MPDP on the activated esters. **d**. Reaction of the aromatic amine DASA precursor polymer with the Meldrum’s acid-based furan adduct to yield the DASA diblock copolymer. **e**. Synthesis of PEG-*b*-PHPMA by PISA.

Current polymersome nanoreactor systems that mimic intercellular communication incorporating out-of-equilibrium feedback loops require medium manipulation through the addition of external chemical fuel^6,10^. To provide a mechanism for more precise feedback loop regulation without requiring addition of external chemical fuel, we designed a system that could be externally manipulated by light. This comprises a nanoreactor that alternates small-molecule semipermeability states by a photoswitch and by a constantly semipermeable nanoreactor that contains a pH-sensitive enzyme. To realize the green-light-triggered nanoreactor (DASA–esterase), a DASA-modified amphiphilic block copolymer was synthesized and self-assembled by the solvent exchange method, while simultaneously encapsulating esterase (polymer synthesis and characterization are described in Extended Data Fig. 1, Supplementary Figs. 2–5 and Supplementary Sections 2–5, and particle characterization in Fig. 2a, Supplementary Figs. 6a–d, 7a–c and 8–10 and Supplementary Section 6). In turn, inherently semipermeable nanoreactors were synthesized using photoinitiated reversible addition-fragmentation chain-transfer polymerization-induced self-assembly (photo-PISA) with in situ encapsulation of urease to yield PISA–urease (Fig. 3a, Extended Data Fig. 1, Supplementary Figs. 6e–h, 7d–f and 11 and Supplementary Sections 2 and 7). Dynamic light scattering revealed nanoparticles with hydrodynamic diameters (*D*_h_) of 207 ± 13 nm in the case of DASA–esterase nanoreactors and *D*_h_ = 362 ± 22 nm for PISA–urease nanoreactors.

### DASA photoswitch-driven enzymatic feedback loop

**Extended Data Fig. 2:**
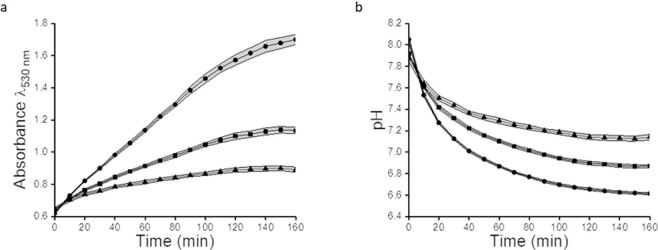
DASA-esterase nanoreactor mediated biocatalytic hydrolysis of ethyl acetate in the presence of MR and under continuous green light irradiation (λ = 530 nm). **a**. Evolution of absorbance over time catalysed by 10 µL (circle), 5 µL (square), and 2 µL (triangle) of 100 times diluted dispersion of DASA-esterase (*n* = 3 technical replicates, mean ± SD). The samples were irradiated by green light (λ = 530 nm) at 1.49 mW∙cm^−2^. The traces at 5 µL of 100 times diluted DASA-esterase dispersion (square) were repeated from Fig. 2D for illustration purposes (*n* = 3 technical replicates, mean ± SD). **b**. pH values calculated from absorbance values in panel a. Source data

**Extended Data Fig. 3:**
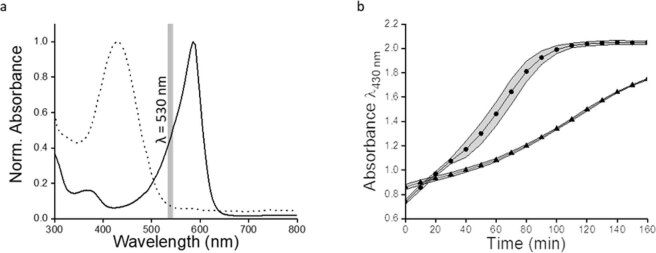
DASA-esterase nanoreactor mediated biocatalytic hydrolysis of ethyl acetate in the presence of bromocresol purple by continuous irradiation of green light (λ = 530 nm). **a**. UV-Vis spectrum of bromocresol purple in acidic (dotted line) and basic (solid line) conditions. The green bar represents green light at λ = 530 nm. **b**. Kinetic absorbance measurements of bromocresol purple protonation at λ = 430 nm catalysed by 10 μL (circle) or 2 μL (triangle) of 100 times diluted DASA-esterase nanoreactors irradiated with green light (λ = 530 nm) (*n* = 3 technical replicates, mean ± SD). The plateau absorbance values coincided with the absorbance of BPH at pH = 1.8. Source data

To generate light-gated negative feedback behaviour by the DASA–esterase nanoreactors, the formation of a product that could compete for the absorption of light was investigated (Fig. 2b). The DASA synthesized in this study was a purple compound that presented an absorbance maximum at *λ* = 550 nm, and absorbed green light at *λ* = 530 nm, causing isomer photoswitching (Fig. 2c). The MR transitioned from a yellow, deprotonated state (*λ*_max_ = 430 nm) to a red, protonated state (*λ*_max_ = 530 nm) below pH 7. Because the absorbance of the red MRH form overlaps with the DASA, as the concentration of MRH increases (due to decreasing pH), it preferentially absorbs more of the incident 530-nm light, thereby acting as a photomask. Thus, DASA–esterase nanoreactors were dispersed in solutions containing ethyl acetate and MR^−^. To study the light-mediated feedback behaviour of these nanoreactors, the dispersions were continuously irradiated with light (*λ* = 530 nm) intensities of 1.49 mW cm^−2^ and 0.76 mW cm^−2^, alongside a dark control with periodic absorbance measurements at *λ* = 530 nm as a measure of MRH photomask accumulation. Under green-light irradiation, a gradual increase of absorbance was observed due to the enzymatic synthesis of acetic acid from ethyl acetate and subsequent formation of MRH (Fig. 2d and Supplementary Fig. 12). Importantly, in the absence of light, this did not occur owing to the polymersome membrane preventing access of substrate (ethyl acetate) to the enzyme, thus confirming the light-gating nature of the DASA nanoreactors between on and off states. Under continuous irradiation, this eventually led to plateaued absorbance, indicating that the biocatalysis had reached equilibrium. Although reactions containing lower MR concentrations reached equivalent MRH absorbance plateau values as that of fully protonated MR at pH 1.8 (Supplementary Fig. 13), reactions containing higher MR concentrations showed increased absorbance throughout the process, correlated to the intensity of light irradiation (Fig. 2d and Supplementary Fig. 12). To understand this phenomenon, we followed the changes in absorbance of the DASA polymer in both organic solution and concentrated aqueous polymersome dispersions after alternation of green-light irradiation and darkness cycles (Fig. 2e and Supplementary Fig. 14). We observed that the rate of isomerization of the purple-coloured DASA to the colourless and more polar cyclopentenone isomer was faster with higher irradiation intensities. Higher light intensities led to greater changes in permeability, which subsequently increased biocatalytic formation of acetic acid over time. In addition, the plateau formation occurred at absorbance values that were proportional to the light irradiation intensity. We hypothesized that the accumulation of MRH masked the penetration of green light, limiting further absorption of light by DASA–esterase, decreasing the permeability of the membrane to the substrate (ethyl acetate) by back-isomerization of the DASA moieties to the less polar triene-enol isomer. Hence, subsequent/further formation of MRH was inhibited. Indeed, higher light intensities penetrated more into the reaction volume and required more MRH photomask formation to quench the gated enzymatic activity of the nanoreactors. To further confirm this hypothesis, we spiked reactions with different concentrations of DASA–esterase (Extended Data Fig. 2). We observed that lower concentrations of DASA–esterase reached plateaus at lower absorbance values, and higher concentrations did not reach plateaus over the same reaction periods. The latter results indicate that both species were absorbing light and that the concentration of accumulated MRH was not sufficient to quench the catalytic activity of DASA–esterase. In addition, we spiked a reaction that had already reached a stationary plateau phase with free esterase, which resulted in a drastic increase in absorbance, demonstrating that this feedback behaviour was not due to self-inhibited enzyme activity at a given pH (Fig. 2f, Supplementary Fig. 15 and Supplementary Section 8). Moreover, control reactions in the presence of a pH pigment that did not overlap in absorbance with DASA–esterase nanoreactors did not display self-inhibitory behaviour (Extended Data Fig. 3 and Supplementary Section 9). Furthermore, control experiments in which free enzyme was irradiated under the same conditions showed no difference in catalytic activity with non-irradiated samples (Supplementary Fig. 16 and Supplementary Section 10), confirming its stability to the irradiation process. This validated the light-driven negative feedback nature of the DASA nanoreactor system, whereby the product formation simultaneously limited its own subsequent production. Importantly, the esterase remained active and the photomasking effect exercised by MRH only interrupted the light-mediated catalysis by DASA–esterase, demonstrating a light-gated negative feedback loop.

### Negative-feedback automodulation of pH-sensitive PISA–urease nanoreactors

To demonstrate the modulation of out-of-equilibrium pH states, a series of experiments were performed to probe the capacity of PISA–urease to produce MR^−^ (Fig. 3a,b). The nanoreactors were immersed in an aqueous solution of acetic acid, MR and urea (starting pH 6.7) and the accumulation of MR^−^ was monitored by kinetic UV–vis spectroscopy (Fig. 3c and Supplementary Section 11). An abrupt decay in absorbance was observed, indicating that urea passively permeated across the membrane of PISA–urease to generate ammonia, leading to MR^−^ formation over time. This process eventually led to a plateau in absorbance, which corresponded to the well-known inactivation of urease enzymes under basic conditions^36^. Importantly, further addition of free urease did not result in absorbance reduction (Fig. 3c). To exclude that this phenomenon was due to the total consumption of substrate fuel (urea), the dispersions were acidified with additional acetic acid (pH 6.8) to generate MRH again (Fig. 3c). Upon acidification, the absorbance decayed over time in a similar fashion to the first cycle, until a plateau was again reached at a similar absorbance. In addition, the pH was calculated using a pH calibration curve for MR (Fig. 3d and Supplementary Sections 11 and 12) confirming the inhibition of PISA–urease nanoreactors at pH ≈ 8.6. This system therefore represented a second pH-mediated negative feedback loop in which the basicity increased (as evidenced by the production of MR^−^) to deactivate further catalysis by PISA–urease nanoreactors. Given that the activity of PISA–urease decreased the concentration of the accumulated MRH photomask, these results highlight the potential to reinitiate the catalytic activity of DASA–esterase when mixing both nanoreactor populations.

### Light-mediated feedback-loop communication between polymersome nanoreactors

**Fig. 4:**
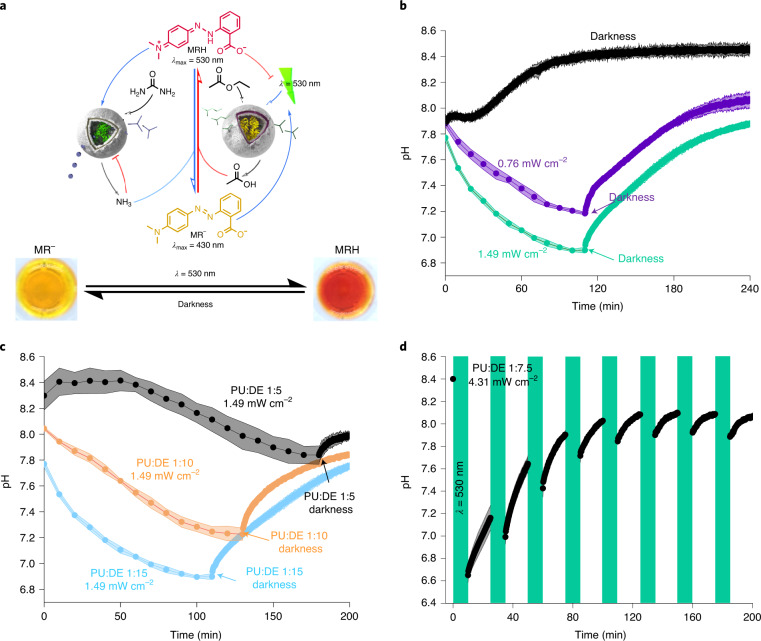
Light-mediated modulation of pH by chemical communication between DASA–esterase and PISA–urease nanoreactors. **a**, Schematic representation of out-of-equilibrium MR protonation by modulation of pH by DASA–esterase and PISA–urease in the presence or absence of light (*λ* = 530 nm). **b**, Monitoring of pH evolution in samples containing a PISA–urease (PU): DASA–esterase (DE) ratio of 1:15 in darkness (black), by continuous irradiation with green light (*λ* = 530 nm) at 0.76 mW cm^−2^ for 110 min followed by darkness (purple), and 1.49 mW cm^−2^ for 110 min followed by darkness (green) (*n* = 3 technical replicates, mean ± s.d.). **c**, Monitoring of pH evolution by continuous irradiation of green light at 1.49 mW cm^−2^ with volume ratios of PU:DE of 1:5 (irradiation for 180 min followed by darkness, black); 1:10 (irradiation for 130 min followed by darkness, orange); 1:15 (irradiation for 110 min followed by darkness, blue) (*n* = 3 technical replicates, mean ± s.d.). The latter was repeated from **b** for illustration purposes. **d**, Monitoring of the evolution of pH by alternating cycles of green light (at 4.31 mW cm^−2^) and darkness. In each cycle, the samples were irradiated for 10 min and the pH was probed for 15 min in darkness (*n* = 3 technical replicates, mean ± s.d.). Source data

In many organisms, circadian rhythm-regulated cell types perform antagonistic enzymatic reactions that control the production rate of metabolites. We sought to mimic aspects of this feature of biological systems with our antagonistic self-regulating nanoreactors to generate controlled fluctuations of pH by light irradiation intensity or alternation of light irradiation and darkness cycles (Fig. 4a).

**Extended Data Fig. 4:**
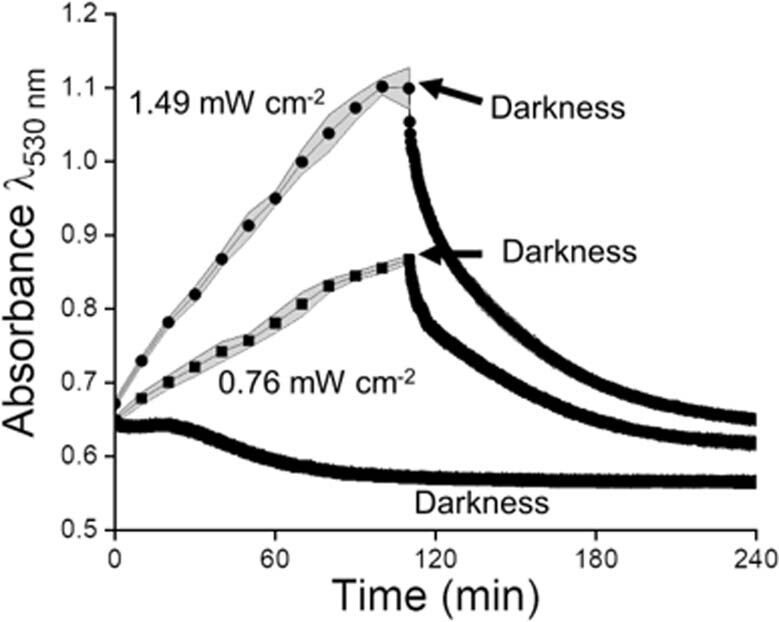
Light-fuelled modulation of pH by chemical communication between DASA-esterase and PISA-urease nanoreactors at different light intensities. Monitoring of absorbance evolution by continuous irradiation of green light at 0 mW∙cm^−2^ (triangle), 0.76 mW∙cm^−2^ for 110 min followed by 0 mW∙cm^−2^ (square), and 1.49 mW∙cm^−2^ for 110 min followed by 0 mW∙cm^−2^ (circle) (*n* = 3 technical replicates, mean ± SD). The corresponding calculated pH values are shown in Fig. 4b. Source data

**Extended Data Fig. 5:**
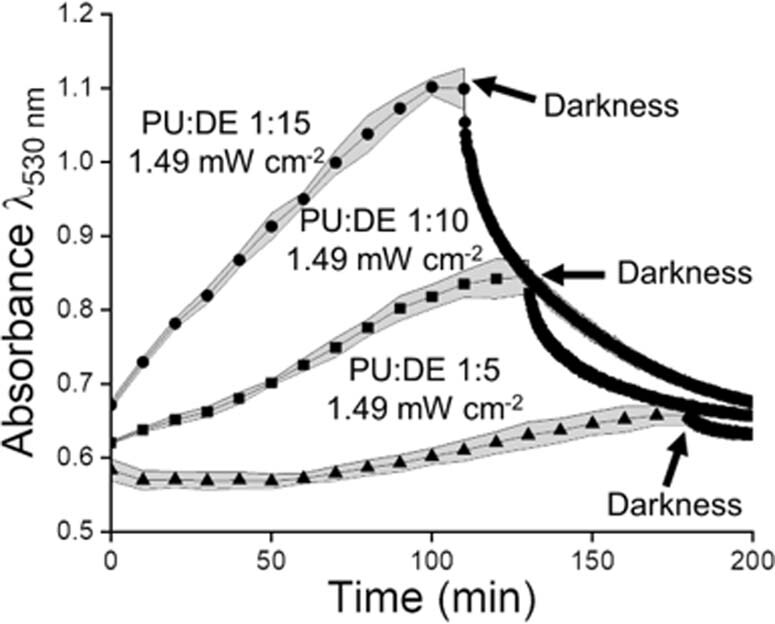
Light-fuelled modulation of pH by chemical communication between DASA-esterase and PISA-urease nanoreactors at different ratios. Monitoring of absorbance evolution by continuous irradiation of green light at 1.49 mW∙cm^−2^ with volume ratios of PISA-urease: DASA-esterase of 1:5 (irradiation for 180 min by irradiation at 0 mW∙cm^−2^) (triangle), 1:10 (irradiation for 130 min followed by 0 mW∙cm^−2^) (square), and 1:15 (irradiation for 110 min followed by irradiation at 0 mW∙cm^−2^) (circle) (*n* = 3 technical replicates, mean ± SD). The latter was repeated from Extended Data Figure 4 for illustration purposes. The corresponding calculated pH values are shown in Fig. 4c. Source data

**Extended Data Fig. 6:**
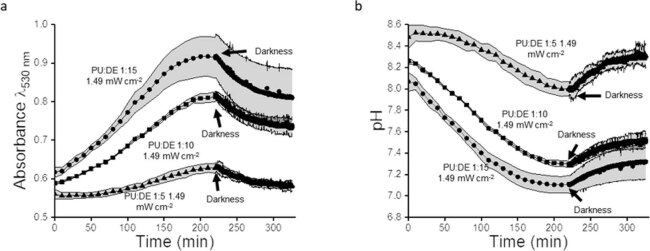
Light-mediated modulation of MR protonation in PBS (10 mM phosphate) by chemical communication between DASA-esterase and PISA-urease nanoreactors. **a**. Monitoring of absorbance evolution by continuous irradiation of green light at 1.49 mW∙cm^−2^ with volume ratios of PU:DE of 1:5 (triangle), 1:10 (square), and 1:15 (circle) (*n* = 3 technical replicates, mean ± SD). In all cases the samples were irradiated by green light (λ = 530 nm) at 1.49 mW∙cm^−2^ for 220 min and in darkness for 105 min. **b**. pH calculation derived from absorbance data in panel **a**. Source data

**Extended Data Fig. 7:**
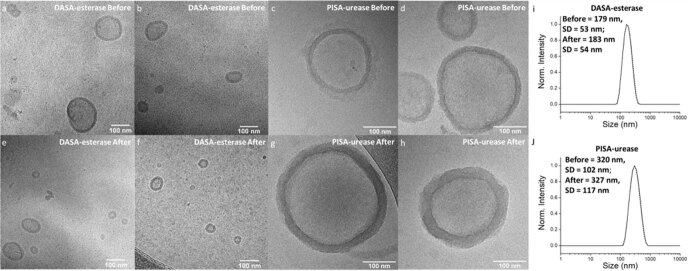
Evaluation of nanoreactor stability to variations in pH. **a, b**. Cryo-TEM micrographs of DASA-esterase nanoreactors at pH = 8.2. **c, d**. Cryo-TEM micrographs of PISA-urease nanoreactors at pH = 8.2. **e, f**. Cryo-TEM micrographs of DASA-esterase nanoreactors after acidification from pH = 8.2 to pH = 6, followed by basification back to pH = 8.2 after 10 min. **g, h**. Cryo-TEM micrographs of PISA-urease nanoreactors after acidification from pH = 8.2 to pH = 6, followed by basification back to pH = 8.2 after 10 min. **i**. DLS measurements before (solid line) and after (dotted line) the oscillation of pH values from panels A-B to E-F. **j**. DLS measurements before (solid line) and after (dotted line) the oscillation of pH values from panels c-d to g-h (*n* = 3 technical replicates; mean). Source data

To generate out-of-equilibrium pH modulation by light, both DASA–esterase and PISA–urease nanoreactors were immersed in an unbuffered aqueous solution containing urea, ethyl acetate and MR (pH 7.8). The dispersions were continuously irradiated with green light at intensities of 1.49 mW cm^−2^ and 0.76 mW cm^−2^ alongside a dark control, and the pH change over time was measured (Fig. 4b, Extended Data Fig. 4 and Supplementary Sections 12 and 13). In the absence of light, the pH increased and remained stable at pH 8.4 over 4 h. This was expected, as DASA–esterase nanoreactors were not semipermeable to the substrate (ethyl acetate) to produce acid, and PISA–urease nanoreactors that were initially active eventually inactivated at higher pH values, in agreement with Fig. 3c,d. In contrast, when the system was irradiated with green light, a gradual decrease in pH was observed, indicating the formation of MRH due to acetic acid production from DASA–esterase. Importantly, higher concentrations of DASA–esterase were required to observe pH changes compared to experiments in the absence of PISA–urease (Figs. 4c and 2d and Extended Data Fig. 4). In addition, lower PISA–urease:DASA–esterase (PU:DE) number ratios generated lower stationary plateau pH values, presumably because the ongoing activation of PISA–urease occurred at pH values closer to the optimum required to compensate for the increased level of esterase activity. In accordance with the previous experiments involving DASA–esterase only, the plateaued pH values were proportional to the irradiation intensities (Fig. 4b). When the light irradiation was withdrawn, the out-of-equilibrium pH values gradually reverted to basic conditions, with higher alkalinization kinetics at lower pH values due to the higher enzymatic activity of PISA–urease (Fig. 4b,c and Extended Data Figs. 4 and 5). Interestingly, in the presence of phosphate buffered saline (PBS; 10 mM phosphate), the reaction kinetics were delayed (Extended Data Fig. 6 and Supplementary Section 13). The system required generation of a higher concentration of products to overcome the buffering capacity of the buffer. In addition, the displayed stationary pH values were similar to unbuffered conditions due to the photomasking effect of MRH and proximity to the optimal pH of the enzymes. Importantly, the individual structures (Extended Data Fig. 7 and Supplementary Section 14) and behaviours of both DASA–esterase and PISA–urease were preserved, highlighting the ability of our system to precisely control pH in out-of-equilibrium systems using visible light.

**Fig. 5:**
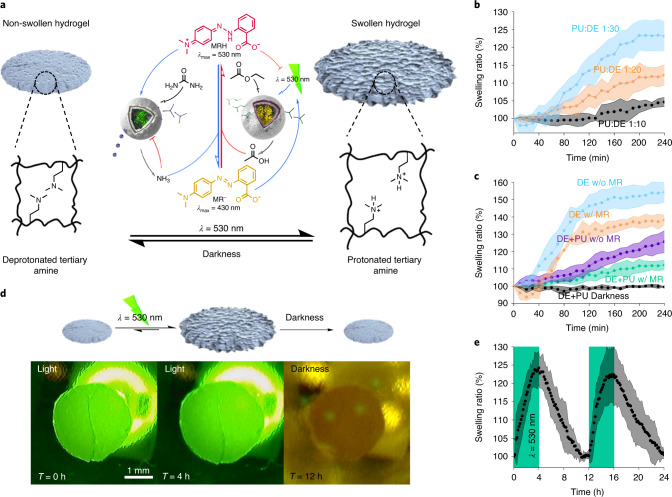
Modulation of hydrogel swelling by communication between antagonistic nanoreactors. **a**, Schematic representation of crosslinked PNIPAAm-*co*-PDMAEMA-*co*-PNBA hydrogel swelling states. The acidification of the medium in the presence of light induces the protonation of the tertiary amine-containing hydrogel to induce swelling. In the absence of light, the recovery of alkaline conditions induces the deprotonation of the hydrogel and subsequent deswelling. **b**, Quantification of the swelling ratio of hydrogels by continuous irradiation of green light (*λ* = 530 nm) at 1.49 mW cm^−2^ with volume ratios of PU:DE of 1:10 (black), 1:20 (orange) and 1:30 (blue) (*n* = 3 technical replicates, mean ± s.d.). **c**, Quantification of swelling ratio of hydrogels by continuous irradiation of green light at 1.49 mW cm^−2^ by DASA–esterase in the absence of MR (blue), in the presence of MR (orange), by DASA–esterase and PISA–urease in the absence of MR (purple), in the presence of MR (green), and DASA–esterase and PISA–urease with MR in darkness (black) (*n* = 3 technical replicates, mean ± s.d.). **d**, Photographs of the reversible hydrogel swelling states, in a 96-well microplate. The images were normalized to the distance corresponding to the bottom of the wells (9 mm). **e**, Reversible swelling and deswelling of hydrogels by alternating green-light irradiation (*λ* = 530 nm) at 1.49 mW cm^−2^ with darkness cycles. The second cycle required further addition of chemical fuel (ethyl acetate and urea) (*n* = 3 technical replicates, mean ± s.d.). Source data

**Extended Data Fig. 8:**
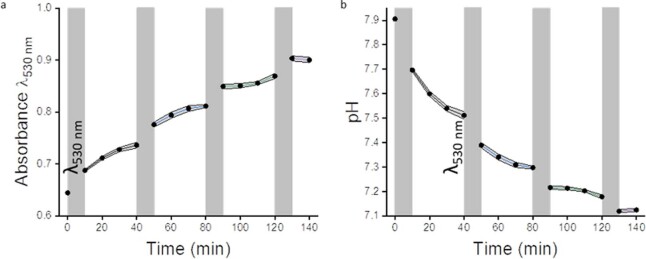
DASA-esterase nanoreactor mediated biocatalytic hydrolysis of ethyl acetate in the presence of MR in alternating light and darkness cycles. **a**. UV-Vis measurements **b**. Calculation of pH from absorbance values. In each cycle, the samples were irradiated with green light at 1.49 mW∙cm^−2^ for 10 min and production of MRH was monitored at 530 nm for 30 min (*n* = 3 technical replicates, mean ± SD). Source data

**Extended Data Fig. 9:**
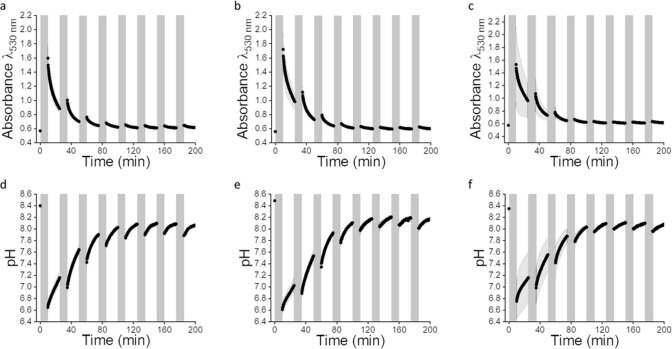
Light-mediated modulation of pH by chemical communication between DASA-esterase and PISA‑urease nanoreactors in the presence of different urea concentrations. UV-Vis measurements monitoring the formation of MRH by alternation of green light at 4.31 mW∙cm^−2^ and darkness. In each cycle, the samples were irradiated for 10 min and the absorbance at λ = 530 nm was probed for 15 min in darkness (*n* = 3, technical replicates, mean ± SD). Reactions were performed in the presence of urea at **a**. 0.41, **b**. 0.83 or **c**. 1.66 mM. Absorbance values were converted to their corresponding pH values and plotted in **d**, **e**, and **f**, respectively. Source data

**Extended Data Fig. 10:**
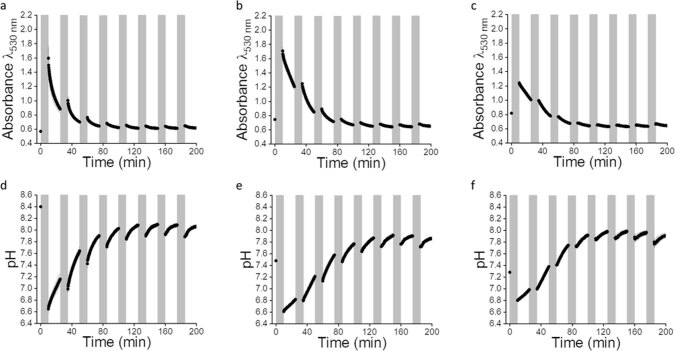
Influence of ammonium acetate concentration on the modulation of medium species by alternating light between DASA-esterase and PISA-urease nanoreactors. UV-Vis measurements monitoring the formation of MRH by alternation of green light at 4.31 mW∙cm^−2^ and darkness. In each cycle, the samples were irradiated for 10 min and the absorbance at λ = 530 nm was probed for 15 min in darkness (*n* = 3, technical replicates, mean ± SD). Reactions were conducted in the presence of ammonium acetate at initial concentrations of **a**. 0, **b**. 10, **c**. ^−1^mM. Absorbance values were converted to their corresponding pH values and were plotted in **d**., **e**., and **f**., respectively. The data represented in panels A and D was repeated from panels B and F from Extended Data Figure 9 for illustration purposes. Source data

In contrast to experiments where the production of acetic acid was not downregulated by the presence of PISA–urease (Fig. 2d), the dispersions containing both DASA–esterase and PISA–urease had the ability to drive back to alkaline pH autonomously in the absence of light irradiation. We thus sought to showcase the modulation of chemical species formation by cycles alternating between green light and dark, allowing the catalytic activity of DASA–esterase to be switched on and off (Extended Data Fig. 8 and Supplementary Section 15). DASA–esterase and PISA–urease were dispersed in solutions of MR, ethyl acetate and urea (pH 8.4) followed by alternating green-light irradiation at 4.31 mW cm^−2^ for periods of 10 min and periods of 15 min in darkness while monitoring the pH fluctuations by UV–vis spectroscopy (Fig. 4d, Supplementary Fig. 17 and Supplementary Section 16). After the first irradiation cycle, DASA–esterase transformed the substrate (ethyl acetate) into acetic acid, producing a decrease in pH to pH 6.6. Throughout the first darkness cycle, the pH gradually increased to pH 7.2 through the acid-mediated activation of PISA–urease, transforming urea into ammonia. Over the following seven cycles, the system displayed alternating shifts of light-induced acid formation and basicity in dark conditions. This can be attributed to reversible switching of the photoresponsive DASA membrane gate. Interestingly, the pH range became gradually more basic over the course of each irradiation and darkness cycle. The presence of residual trypsin from the synthesis of the DASA–esterase polymersomes was excluded as a possible reason for the observed decay in oscillatory capacity, as no notable changes were observed in the presence of a trypsin inhibitor (Supplementary Fig. 18 and Supplementary Section 16). Moreover, the enzymes were shown to be stable to the high irradiation intensity of the experiment (Supplementary Fig. 19 and Supplementary Section 10). Beyond fuel consumption, the loss in light-induced esterase activity may be explained by the loss of enzymatic activity due to a number of factors, including the build-up of ammonium acetate buffer salts in the confined reaction volume. We thus performed reactions containing different concentrations of urea (Extended Data Fig. 9 and Supplementary Section 16). Over the cycles of alternating light irradiation and darkness, we observed that reactions containing lower concentrations of urea displayed higher changes in pH oscillation. Over time, these values decayed to similar values as experiments with higher concentrations of urea. We attribute this to a lower accumulation of ammonium acetate salts that buffered the system over time. To confirm this hypothesis, reactions containing predissolved ammonium acetate were performed (Extended Data Fig. 10 and Supplementary Section 16). Over the irradiation cycles, the variations in pH were less pronounced than in reactions that were initially unbuffered, and tended to stabilize close to the more acidic initial buffer pH values.

Overall, the communication between DASA–esterase and PISA–urease nanoreactors was governed by the two independent negative feedback loops, as described above, and by two positive feedback loops. Light activated the production of acetic acid and MRH, which activated PISA–urease. The production of ammonia by PISA–urease generated MR^−^, allowing light to stimulate the catalysis of more acetic acid and MRH. This process constituted a first positive feedback loop. In turn, the production of ammonia, by PISA–urease, produced MR^−^, which allowed light to stimulate DASA–esterase and produce more acetic acid and MRH. The shift in pH further activated the production of ammonia by PISA–urease, thereby constituting a second positive feedback loop (Fig. 1c). Conceptually, this light-powered process mimics aspects of the complex circadian rhythm feedback loops observed in living organisms.

### Out-of-equilibrium chemomechanical coupling of polymersome nanoreactors in swellable hydrogels

In multicellular organisms, the modulation of metabolite concentrations can reversibly control the physical state of tissues and organs. Inspired by such processes, we investigated how the light-mediated modulation of pH states by DASA–esterase and PISA–urease nanoreactors could be coupled to the swelling of a hydrogel as a rudimentary representation of how chemomechanical work could be generated by an out-of-equilibrium nanoreactor network. For this purpose, we synthesized hydrogels of chemically crosslinked poly(*N*-isopropylacrylamide)-*co*-poly(2-(dimethylamino)ethyl methacrylate)-*co*-poly(Nile blue acrylamide) (PNIPAAm-*co*-PDMAEMA-*co*-PNBA) containing tertiary amines. These become protonated at acidic pH (p*K*_a_ ≈ 7.5)^37^ and swell (Fig. 5a). Nile blue was incorporated to facilitate image contrast.

The circular gels (*d* = 2 mm) were immersed in dispersions containing the two nanoreactor populations at different number ratios as well as MR, ethyl acetate and urea. After equilibration for 1 h, the samples were irradiated with green light (1.49 mW cm^−2^), and the hydrogel swelling ratio was monitored over 4 h using a dermatoscope (Fig. 5b, Supplementary Fig. 20a and Supplementary Section 17). Upon light irradiation, DASA–esterase nanoreactors became semipermeable to the substrate (ethyl acetate), allowing the catalytic formation of acetic acid, which simultaneously protonated the MR^−^ reporter and pH-responsive hydrogel, resulting in MRH formation and hydrogel swelling, respectively. Eventually, the swelling process reached a plateau, indicating equilibrium of the hydrogels with the bulk solution pH. Importantly, the out-of-equilibrium swelling states were proportional to the PU:DE number ratio. In accordance with spectroscopic measurements of pH (Fig. 4c), lower PU:DE ratios generated lower stationary pH values, which translated into higher protonation and swelling states of the hydrogels.

To test the role of MRH formation as a modulator of hydrogel swelling, we immersed the hydrogels in dispersions containing fixed concentrations of the two nanoreactor populations and irradiated them with green light at 1.49 mW cm^−2^ in the presence and absence of MR^−^ alongside a control in darkness (Fig. 5c and Supplementary Fig. 20b). Although hydrogel swelling was not observed in darkness, higher swelling rates were observed in samples that did not contain MR. The results indicate that the formation of acid and subsequent protonation of the hydrogel were limited by the negative feedback generated by accumulation of the photomask MRH. In addition, similar behaviour was observed in experiments where DASA–esterase was present and PISA–urease was absent, which additionally generated higher swelling states (Fig. 5c and Supplementary Fig. 20b). To demonstrate that the hydrogel swelling was an out-of-equilibrium state, the samples were investigated after withdrawal of green light. After removing the light source, the enzymatic production of acetic acid was interrupted, and PISA–urease nanoreactors dominated the modulation of pH by accumulation of basic ammonia. This caused progressive deprotonation of the hydrogel tertiary amines, leading to gradual deswelling. The swelling could be reinitiated by the addition of substrate fuel (ethyl acetate and urea) and further irradiation with green light for another 4 h, demonstrating the reversible nature of this ternary system. Overall, the changes in swelling state of the hydrogels were consistent with the changes in pH observed in spectroscopic measurements (Fig. 4). These results confirm that the chemical species generated during the out-of-equilibrium communication of the antagonistic nanoreactors can be employed for chemomechanical work, thereby modulating the physical properties of an auxiliary hydrogel, akin to solid tissue matrices.

## Conclusions and future perspectives

Bioinspired out-of-equilibrium materials that require constant energy to maintain activated states of organization or chemical composition provide a step to the next generation of molecular materials with adaptive, autonomous and intelligent behaviour^38–49^. Cells require a constant energy supply to remain in an out-of-equilibrium state and perform functions that dictate their survival. In this Article we showcase how a ternary system of polymersome nanoreactors and a hydrogel could be employed to mimic complex biological communication processes and self-regulate their behaviour through positive and negative feedback loops. These nanoreactors, which are in a dormant equilibrium state in dark alkaline conditions, shift to an out-of-equilibrium state when irradiated with light. Importantly, the system is composed of three delayed chemical modulators. First, the permeability of DASA–esterase to its substrate fuel is modulated by the presence of light. Second, the pH of the bulk solution evolves in a delayed manner through the accumulation of acid and alkali by DASA–esterase and PISA–urease, respectively. Third, a delayed swelling and deswelling of the hydrogel occurs in response to the modulation of pH in the bulk solution. With further optimization, such systems could be adapted for novel and diverse applications, including artificial organelles, protocells or soft robotic prototissues.

So far, most reports employing enzymes in out-of-equilibrium conditions require a constant supply of chemical fuel to maintain their output chemical signals, thus hindering their application in closed materials^1^. In contrast, the work described here showcases a system that can be shifted on and off by an external macroscopic stimulus, until the chemical fuel is consumed. Another important achievement of this work is successfully deciphering a method to modulate the pH of a medium with light-controlled enzymes. Such approaches can be employed as a powerful tool to control a wide spectrum of catalysts as, in many cases, their turnover can be modulated by approximation to their optimal pH. Towards the goal of replicating living biological materials, understanding the underlying concepts behind the molecular mechanisms that drive biological functions can provide key insights into the fabrication of novel out-of-equilibrium systems and materials. In this regard, polymersome nanoreactors mimic the compartmentalizing nature of biological membranes, and represent a robust platform for the creation of complex functional materials with life-like properties. In addition, the system was capable of performing in biologically relevant buffered conditions, opening an avenue to light-controlled inter-nanoreactor signaling and nanoreactor/living cell chemical communication. The future outlook for this work will expand on employing light-responsive nanoreactors with a broader range of enzymes to animate materials capable of autonomous motion, as well as self-organization, self-actuation and adaptive behaviour.

## Methods

### Materials and equipment

Poly(ethylene glycol) 4-cyano-4-(phenylcarbonothioylthio)pentanoate (*M*_n_ ≈ 2,000 g mol^−1^, determined by supplier), 2,6-lutidine (98%), pentafluorophenol (≥99%), acryloyl chloride (≥97%, contains ~400 ppm phenothiazine as stabilizer), butyl acrylate (≥99%, contains 10–60 ppm monomethyl ether hydroquinone as inhibitor), 1,4-dioxane (99.8%), methanol (99.8%), ethyl acetate (>99.5%) (Sudan Blue II (98%), 2,2-dimethyl-1,3-dioxane-4,6-dione (98%), furfural (99%), *p*-iodoanisol (98%), 1,3-diaminopropane (≥99%), copper (I) bromide (≥99.5%), triethylamine (≥99.5%), ammonium sulfate (≥99%), sodium bicarbonate (>99.5%), esterase from porcine liver (lyophilized, powder, slightly beige, ≥50 U mg^−1^), urease from *Canavalia ensiformis* (Jack bean), Type IX (powder, 50,000–100,000 U g^−1^ solid), trypsin from bovine pancreas (Type I, ~10,000 BAEE U mg^−1^ protein), 2-(dimethylamino)ethyl methacrylate (98%), methyl red (crystalline), Nile blue acrylamide, Rhodamine B isothiocyanate (mixed isomers, BioReagent, suitable for protein labelling), ammonium acetate (>97%), bromocresol purple sodium salt (indicator grade dye), aprotinin from bovine lung (lyophilized powder, 3–8 TIU mg^−1^ solid), DMSO and chloroform-D1 (deuteration degree min. 99.8% for NMR spectroscopy) were purchased from Sigma Aldrich and were used as received. *N*-isopropyl acrylamide (97%) was purchased from Sigma Aldrich and recrystallized from hexane. 2-Hydroxypropyl methacrylate was purchased from Sigma Aldrich and was purified by silica column chromatography, employing *n*-hexane:ethyl acetate 90:10. Urea (powder, BioReagent for molecular biology, suitable for cell culture) was purchased from Sigma Aldrich and recrystallized from ethanol. Dichloromethane (DCM; 99.9%), ethanol (99.9%), tetrahydrofuran (THF; 99.9%), dimethylformamide (DMF; >99.8%), magnesium sulfate (99.6%), sodium sulfate (99.8%), sodium hydroxide (≥99%) and sodium chloride (≥99%) were purchased from VWR Chemicals. Lithium phenyl(2,4,6-trimethylbenzoyl)phosphinate (>98%) was purchased from TCI Chemicals UK. Dulbecco’s PBS was purchased from Gibco. Azobisisobutyronitrile (AIBN) was purchased from Molekula and recrystallized from methanol. Biobeads S-X3 (600–14,000 g mol^−1^ range) were purchased from Bio-Rad Laboratories. Alexa Fluor 647 C2 maleimide was purchased from Thermo Fisher Scientific. Ultrapure water was obtained from a Triple Red Avidity Science Duo at 18.2 mΩ cm^−1^.

UV–vis spectroscopy was carried out on a Molecular Devices Spectramax M5 plate reader.

Polymer molecular weight (*M*_n, GPC_) and dispersity (*Ð*) were measured using a 1260 Infinity II GPC MDS system (refractive index detection only) equipped with a PSS GRAM guard column (8 × 50 mm, 10 µm) and two PSS GRAM linear columns (8 × 300 mm, 10 µm, 500–1,000,000 Da) and utilizing HPLC-grade DMF (containing 0.075 wt% LiBr) at 40 °C as eluent (flow rate of 1 ml min^−1^). Molecular-weight calibration was performed using near-monodisperse poly(methyl methacrylate) standards (EasiVial, Agilent).

Dynamic light scattering was used to determine the hydrodynamic diameter (*D*_h_) and polydispersity of the nanoreactors in ultrapure water, and was measured using a Zetasizer Nano ZS device. The scattering angle was fixed at 173°. Data processing was carried out using cumulant analysis of the experimental correlation function, and the Stokes−Einstein equation was used to calculate the hydrodynamic radii. All solutions were analysed using disposable poly(styrene) ZEN0040 microcuvettes.

Light stimulation of the DASA was performed by a Teleopto LAD-1 light-emitting diode (LED) array driver powering a LEDA-G (*λ* = 530 nm) LED array. Photopolymerization to yield the PISA–urease nanoreactors was performed by a Teleopto LAD-1 LED array driver powering a LEDA-V (*λ* = 405 nm) LED array.

Attenuated total reflection Fourier-transform infrared spectra were recorded on a Perkin Elmer Spectrum 100 FT-IR spectrometer equipped with a diamond crystal insert.

^1^H NMR spectra were recorded on a Bruker Avance III HD 600 MHz device at 298 K, employing the standard Bruker pulse programs and parameter sets. For diffusion-edited ^1^H NMR spectra, 40% gradient strengths were applied to selectively suppress the signals of low-molecular-weight species. The proton signals were referenced internally with residual resonances of the solvent. ^19^F NMR was recorded on a Jeol 400-MHz spectrometer. All measurements were carried out in CDCl_3_.

pH measurements were performed with an InLab micro pH-electrode connected to a Mettler Toledo FiveEasy plus pH meter.

Photographic imaging of the hydrogels in 96-well microplates was performed with a fully polarized Firefly DE300 Polarizing Handheld USB digital dermatoscope. Images were recorded every 5 min. Measurement of object distances was performed with ImageJ (1.52n, Wayne Rasband, National Institutes of Health, USA, Java 1.9.0_172 (64-bit)).

For cryogenic transmission electron microscopy (cryo-TEM) for Supplementary Fig. 6 and Extended Data Fig. 7a,b,e,f, 3.0-μl samples were applied to either glow-discharged Quantifoil R2/2 holey carbon grids (Quantifoil Micro Tools) or 400-mesh Cu-grids (TAAB Laboratories Equipment) covered with an additional thin continuous carbon film. Frozen-hydrated specimens were prepared with an automatic plunge freezer FEI Vitrobot (Thermo Fisher Scientific), operated at 16 °C and 100% relative humidity. The samples were incubated for 10 s on the grids, blotted for 3–4 s and plunged into liquid ethane. The cryo-specimens were transferred to a JEOL JEM-2100f transmission electron microscope (JEOL) operated at 200 kV. Images were recorded using a TVIPS TemCam-XF416 CMOS camera (Tietz Video and Image Processing Systems). Brightness and contrast correction of the images were performed using ImageJ, as well as the measurement of object distances. For Extended Data Fig. 7c,d,g,h, a 4-µl aliquot of the samples was adsorbed onto a holey carbon-coated grid (Lacey), blotted with Whatman 1 filter paper and vitrified into liquid ethane at −180 °C using a Leica GP2 plunger (Leica Microsystems). Frozen grids were transferred onto a Talos L120C electron microscope (FEI) using a Gatan 626 cryo-holder (GATAN). Electron micrographs were recorded at an accelerating voltage of 120 kV using a low-dose system (40 e^−^/Å^2^) while keeping the sample at −175 °C. Defocus values were −2 to 3 µm. Micrographs were recorded on a 4k × 4k Ceta CMOS camera.

Fluorescence correlation spectroscopy (FCS) was performed using a commercial LSM 880 system (Carl Zeiss). The incubation chamber was held at 37 °C. A 561-nm- or 633-nm-wavelength excitation source (HeNe laser) was used in combination with an appropriate filter set and a ×40 C-Apochromat water immersion objective (numeric aperture of 1.2). Five-microlitre droplets of sample were pipetted onto glass-bottom ibidi eight-well plates (80827, ibidi). The laser focus was then moved 200 µm above the glass plate to conduct the FCS measurements. Sulforhodamine B (SRB) in PBS was used to calibrate the beam waist at 561-nm excitation (*D* = 4.14 × 10^−6^ cm^2^ s^−1^ (SRB) at 25 °C was used to calculate *D* = 5.54 × 10^−6^ cm^2^ s^−1^ at 37 °C)^50^. Alexa Fluor 647 (AF647) in PBS was used to calibrate the beam waist at 633-nm excitation (*D* = 3.3 × 10^−6^ cm^2^ s^−1^ at 25 °C, which is *D* = 4.42 × 10^−6^ cm^2^ s^−1^ at 37 °C). A total of 30 × 5-s intensity traces were measured per sample; the average correlation curves across the whole measurements (150 s) are given in the figures. ZEN software (Carl Zeiss) automatically autocorrelated the data and, after exporting, the data were fitted using PyCorrfit program 1.1.6 (ref. ^51^), employing one-component fits:

$${G}_{1{\rm{comp}}}{\left( \tau \right)} = {\left( {1 + {\frac{T}{{1 - T}}}{\rm{e}}^{\frac{{ - \tau }}{{\tau _{\rm{trip}}}}}} \right)} \times {\frac{1}{{N \times \left( {1 + \frac{\tau }{{\tau _{\rm{D}}}}} \right) \times {\sqrt {1 + \frac{\tau }{{{\rm{SP}}^2\tau _{\rm{D}}}}}}}}}$$

The height-to-waist ratio (structural parameter, SP) was fixed to 5. *τ*_trip_ is the triplet time with corresponding triplet fraction *T*, *τ*_D_ is the diffusion time, and *N* is the number of diffusing species in the confocal volume. A solution of SRB in PBS was measured to calibrate the *x*–*y* dimension of the confocal volume (${\omega _{xy}^2}$). Using the obtained diffusion times (*τ*_D_) from above, the diffusion coefficients (*D*) were subsequently obtained for each unknown sample:

$${D} = {\frac{{\omega _{xy}^2}}{{4\tau _{\rm{D}}}}}$$

The Einstein–Stokes equation was subsequently used to calculate the hydrodynamic radii (*R*_h_) using the obtained diffusion coefficients (*D*) from above. Cargo numbers per polymersome were calculated by dividing the counts per particle (CPP) obtained for loaded polymersomes by the value obtained for free labelled cargo.

To calculate the encapsulation efficiency, DASA–esterase was formulated by loading 525 nM esterase-AF647 (85 µg ml^−1^), and subsequent trypsinization. This lower concentration compared to the other experiments was required due to the inability to purify the sample from non-encapsulated, trypsinized fragments. After performing FCS measurements, the data were analysed using two-component fits:

$$\begin{array}{l}{G}_{2{\rm{comp}}}{\left( \tau \right)} = {\left( {1 + \frac{T}{{1 - T}}{\rm{e}}^{\frac{{ - \tau }}{{\tau _{\rm{trip}}}}}} \right)} \times {\frac{1}{N}} \\\times {\left[ {\frac{{F_1}}{{\left( {1 + \frac{\tau }{{\tau _1}}} \right) \times {\sqrt {1 + \frac{\tau }{{{\rm{SP}}^2\tau _1}}}}}} + {\frac{{1 - F_1}}{{\left( {1 + \frac{\tau }{{\tau _2}}} \right) \times \sqrt {1 + \frac{\tau }{{{\rm{SP}}^2\tau _2}}}} }}} \right]}\end{array}$$

where *τ*_1_ and *τ*_2_ are the diffusion times of the two fractions *F*_1_ (free esterase-AF647) and *F*_2_ (particle fraction) and *N* is the total number of diffusing species in the confocal volume. A triplet fraction *T* with corresponding triplet time *τ*_trip_ was included and fixed between 1 and 10 µs, and the structural parameter SP, which is the ratio of height to width of the confocal volume, was set to 5. In this experimental set-up without purification, *F*_*2*_ × 100 corresponds to the encapsulation efficiency. A control mixture of free esterase-AF647 with unloaded DASA polymersomes gave minimal *F*_*2*_, which was subtracted from the value for DASA–esterase-AF647 to yield an encapsulation efficiency of 7.5%.

### Synthesis of pentafluorophenyl acrylate

This compound was synthesized following a procedure published by Théato and colleagues (Extended Data Fig. 1a)^52^.

### Synthesis of poly(ethylene glycol)-*b*-(poly(butyl acrylate)-*co*-poly(pentafluorophenyl acrylate))

Chain extension by reversible addition-fragmentation chain-transfer radical polymerization was performed on the macro chain-transfer agent (macroCTA) poly(ethylene glycol) 4-cyano-4-(phenylcarbonothioylthio) pentanoate (PEG-CPADB) (**1**), yielding an amphiphilic diblock copolymer with a randomly distributed activated ester (Extended Data Fig. 1a). AIBN (1.0 mg, 6.09 μmol) and PEG-CPADB (97.4 mg, 48.7 μmol) were dissolved together in 0.77 ml of 1,4-dioxane. In parallel, butyl acrylate (BA) was purified from monomethyl ether hydroquinone by perfusion through a basic aluminium oxide plug. Then, pentafluorophenyl acrylate (PFPA; 52.0 μl, 72.5 mg, 0.3 mmol) and the purified BA (0.39 ml, 0.35 g, 2.74 mmol) were added to the initiator and macroCTA solution. Oxygen was removed from the resulting solution by bubbling with argon flow for 1 h. The stoichiometry I:CTA:M was 1:8:500 and the monomer ratio BA:PFPA was 9:1. The reaction was initiated by exposing the solution to 90 °C in an argon atmosphere. The reaction was ended after 3 h by exposure to atmospheric oxygen. The polymer was purified by precipitation in 60:40 vol/vol methanol:water. The resulting suspension was centrifuged at 7,000*g* for 15 min at room temperature (Eppendorf 5430/5430R, F-35-6-30 rotor). To remove the remaining non-polymerized monomer, the pellet was dissolved in 2 ml of THF and an end of spatula of Sudan Blue II was added as a small-molecule indicator for preparative size exclusion chromatography (SEC). SEC was performed using Biobeads S-X3 (600–14,000 g mol^−1^ range) as the stationary phase and distilled THF as the mobile phase. The elution volume before the appearance of the blue small-molecule indicator fraction was collected and concentrated in vacuo. After further drying the polymer in a vacuum oven at 40 °C overnight, 360 mg (69% yield) was collected (Supplementary Fig. 5a).

### Removal of the chain-transfer agent end group from PEG-*b*-(PBA-*co*-PPFPA)

The chain-transfer agent was removed from PEG-*b*-(PBA-*co*-PPFPA) (**2**) following a procedure reported by Perrier et al. (Extended Data Fig. 1b)^53^. The polymer (360 mg) was dissolved with 200 mg (1.2 mmol) of AIBN in DMF (3 ml) in a round-bottom flask. The solution was bubbled for 1 h with N_2_ flow and posteriorly sealed under a N_2_ atmosphere. The reaction was initiated by transferring the solution to an 80 °C oil bath and carried for 4 h. Then, the polymer was purified by SEC. For this purpose, an end of spatula of Sudan Blue II was added as an indicator for the small-molecule fraction, and Biobeads S-X3 (600–14,000 g mol^−1^ range) was employed as the stationary phase, using distilled THF as eluent. The organic solution was collected prior to the appearance of the Sudan Blue II fraction and was concentrated in vacuo. The polymer, was further dried in a vacuum oven overnight at 40 °C (Supplementary Figs. 2a, 3a and 4).

### Synthesis of the second generation DASA donor precursor *N*-(4-methoxyphenyl)−1,3-diaminopropane

The compound was synthesized following a procedure published by Han et al. (Extended Data Fig. 1c)^54^.

### Synthesis of aromatic amine DASA precursor diblock copolymer (3)

The AIBN-capped PEG-*b*-(PBA-*co*-PPFPA) was dissolved in anhydrous DMF (3 ml) together with *N*-(4-methoxyphenyl)-1,3-diaminopropane (100 mg, 5.5 × 10^−1^ mmol) and triethylamine (0.10 ml, 0.73 mg, 1 mmol) (Extended Data Scheme 1c). The solution was degassed by bubbling with N_2_ flow for 30 min. The reaction was carried out at 60 °C for 12 h. Subsequently, the polymer was purified by SEC, employing an end of spatula of Sudan Blue II as an indicator for the small-molecule fraction. The reaction mixture was poured onto Biobeads S-X3 (600–14,000 g mol^−1^ range) and the polymer was eluted with distilled THF. The volume before the appearance of the blue small-molecule fraction was collected, concentrated in vacuo, and dried in a vacuum oven at 40 °C overnight (Supplementary Figs. 3b, 4 and 5b,c).

### Synthesis of Meldrum’s acid-based furan adduct

This compound was synthesized according to a procedure described by Read de Alaniz and co-workers (Extended Data Fig. 1d)^55^.

### Modification of aromatic amine DASA precursor polymer to yield the DASA polymer

The secondary aromatic amine on the diblock copolymer (**3**) was reacted with Meldrum’s acid-based furan adduct to yield the DASA diblock copolymer (Extended Data Fig. 1d). In short, 100 mg (1.2 × 10^1^ μmol) of the polymer was dissolved in a solution of Meldrum’s acid-based furan adduct (9 × 10^−1^ M) in a round-bottom flask. The solution was capped with a rubber septum and allowed to stir at room temperature for seven days. The polymer was then purified from the excess of furan adduct by SEC. Biobeads S-X3 (600–14,000 g mol^−1^ range) was employed as the stationary phase and distilled THF as the mobile phase. In this case, the addition of small-molecule indicator was not required, as both the furan adduct and the polymer presented distinctive colours. Finally, the polymer was dried in a vacuum oven at 40 °C overnight (Supplementary Figs. 2a, 4 and 5d).

### Preparation of DASA–esterase nanoreactors

Unless otherwise stated, 1 mg (1.1 × 10^−1^ μmol) of the DASA diblock copolymer was weighted in an HPLC vial and dissolved in 0.1 ml of 1,4-dioxane. The self-assembly was conducted in a dimly lit room to favour the triene-enol form of the DASA—its more hydrophobic and colourful state. In a different HPLC vial, 1 mg of esterase from porcine liver (5.95 nmol) was dissolved in 1 ml of ultrapure water and equipped with a magnetic stir bar. The solution was stirred at level 2 of a Stuart UC151 hot plate, and the polymer solution was added gradually in 10-μl steps. The resulting dispersion was allowed to stir for another 5 min. To digest the non-encapsulated esterase, the dispersion was transferred to a third HPLC vial containing 0.5 mg of trypsin (21 nmol) and incubated at 37 °C overnight (Supplementary Fig. 8). Finally, the sample was stored at 4 °C.

To facilitate cryo-TEM imaging, 2 mg (2.2 × 10^−1^ μmol) of the of the DASA block copolymer was used instead of 1 mg (Supplementary Fig. 6a–d).

### Synthesis of urease-loaded polymersomes by PISA

#### Synthesis of PEG_113_-CDTPA

CDTPA (0.97 g, 2.4 mmol), PEG_113_ (*M*_n_ = 5,000 g mol^−1^; 6 g, 1.2 mmol) and DCM (20 ml) were charged to a round-bottom flask equipped with a stir bar. A solution of *N*,*N*′-dicyclohexylcarbodiimide (DCC) (0.5 g, 2.4 mmol) and 4-dimethylaminopyridine (DMAP) (0.029 g, 0.24 mmol) in DCM (10 ml) was added dropwise to the reaction flask, which was maintained at 0 °C using an ice-water bath. The flask was subsequently sealed with a rubber septum and purged with N_2_ for 30 min at 0 °C. The esterification reaction was allowed to proceed with stirring at room temperature for 48 h in the dark. A yellow polymer was collected by three repeated precipitation/centrifugation (7,000*g* for 5 min) cycles of the reaction mixture in cold diethyl ether (Supplementary Fig. 2b).

#### Synthesis of urease-loaded PEG_113_-*b*-PHPMA polymersomes

PEG_113_-CDTPA (5.67 mg, 1.05 µmol), 2-hydroxypropyl methacrylate (HPMA) (60.3 mg, 56.6 µl, 4.18 × 10^−4^ mmol) and 441.2 µl of Jack Bean urease (10 mg ml^−1^) in PBS was added to a 2.5-ml crimp vial equipped with a magnetic stir bar (Extended Data Fig. 1e). The vial was sealed and degassed by bubbling with N_2_ flow for 30 min. The vial was then irradiated using an LED array (*λ*_max_ = 405 nm, *I* ~ 10 mW cm^−2^) for 3 h with magnetic stirring. To purify the polymersome samples, the turbid solution was removed from the light source, diluted 10 times with ddH_2_O and spun at 16,000*g* for 10 min. The supernatant was carefully removed and the polymersome pellet resuspended in 5 ml of fresh ultrapure water. The centrifugation process was repeated an additional two times to obtain the purified urease-loaded PEG-*b*-PHPMA polymersomes (PISA–urease; Supplementary Figs. 2b and 6e–h).

### Fluorescent dye functionalization of enzymes and preparation of samples for FCS measurements

#### Functionalization of esterase and urease with Rhodamine B isothiocyanate

Rhodamine B isothiocyanate (0.33 mg, 6.1 × 10^−1^ μmol) was weighted in a 1.5-ml screw HPLC vial and dissolved in 33 μl of DMSO. In a separate HPLC vial, either 2 mg (11.9 nmol) of esterase or 2 mg (3.7 nmol) of urease was dissolved in 400 μl of ultrapure water and equipped with a magnetic stirrer bar. The organic solution was transferred to the aqueous protein solution. The vial was covered in aluminium foil and the reaction was allowed to proceed for 2 h while stirring. The labelled esterase (RhB–esterase) was purified by SEC, using two sequential PD Minitrap G-25 and PD Miditrap G-25 (GE Healthcare) devices. In the case of urease, the solution was dialysed against 1 l of ultrapure water using a Float-A-lyzer G2 dialysis device (molecular weight cutoff (MWCO) = 100,000 g mol^−1^). The elution volume was exchanged once per day throughout four days. In both cases, the labelled protein powders were concentrated by lyophilization (RhB–esterase, 1.3 mg, 65% yield; RhB–urease, 1.8 mg, 90% yield; Supplementary Figs. 8b,c, 11b and 18a).

#### Functionalization of esterase with Alexa Fluor 647-maleimide

Alexa Fluor 647-maleimide (0.1 mg, 8 × 10^−1^ μmol) was dissolved in 10 μl of DMSO and added to a solution of 1 mg (5.95 nmol) of esterase in ultrapure water while stirring with a magnetic stir bar in a HPLC vial. The vial was covered in aluminium foil and the reaction was allowed to proceed for 2 h. The labelled esterase (AF647-esterase) was purified by dialysis using a Float-A-lyzer G2 system (MWCO = 100,000 g mol^−1^) against 1 l of ultrapure water. The eluent was changed once per day over three days. The protein was further purified by SEC, using a PD-10 column (Cytiva) containing Sephadex G-25M. A 2.5-ml volume of the dialysed protein was added to the column and 3.5 ml were collected. The resulting concentration was 85 μg ml^−1^ (525 nM; 31% yield) for FCS. The solution was kept refrigerated at 4 °C (Supplementary Fig. 9).

#### Preparation of trypsinized RhB–esterase

Aqueous solutions of RhB–esterase (1 mg ml^−1^) were incubated with trypsin (0.3 mg ml^−1^) at 35 °C for 12 h to induce protein digestion (Supplementary Fig. 8b,c).

#### PISA polymersomes loaded with rhodamine B-modified urease

To generate PISA–RhB–urease, a solution of 9 mg ml^−1^ of commercial urease and 1 mg ml^−1^ of RhB–urease was prepared and all other reaction conditions for the synthesis of PISA–urease were employed (Supplementary Fig. 11).

### Photoswitching and thermal recovery measurements of the DASA polymer

#### Photoswitching of the DASA polymer in THF

A solution of 24 µg ml^−1^ of the DASA polymer in THF was introduced in a 1-ml quartz cuvette. UV–vis measurements were performed at 530 nm on the cuvette holder of a Spectramax M5 device (Molecular Devices). After an initial measurement, the samples were irradiated for 30 s at 530 nm at intensities of either 0.23 mW cm^−2^, 0.76 mW cm^−2^ or 1.49 mW cm^−2^. The thermal recovery was monitored over 15 min every 10 s. This process was repeated three times. The experiments were carried out in triplicate, and the values of the three cycles were normalized to the maximum absorbance of the first cycle. Finally, the data were laid in sequence (Supplementary Fig. 14).

#### Photoswitching of aqueous DASA polymer dispersions

The organic solution was substituted by a dispersion of 0.05 mg ml^−1^ of the DASA polymer in ultrapure water. A 150-μl volume of this dispersion was transferred to a Corning 96-well half-area microplate (poly(styrene), non-binding). Data acquisition and treatment were performed in the same manner as for the polymers in organic solution. In this case, the samples were irradiated at 0.76 mW cm^−2^, 1.49 mW cm^−2^ or 2.12 mW cm^−2^ for 10 min, and the thermal recovery was monitored over 30 min every 10 s (Fig. 2e).

### Determination of esterase activity of DASA–esterase nanoreactors

Unless otherwise stated, 5 μl of 100 times diluted DASA–esterase dispersion was introduced into 295 μl of an aqueous solution of MR (7.4 × 10^−1^ mM) and saturated ethyl acetate (pH 7.8) contained in a Corning 96-well microplate (poly(styrene), non-binding). The plate was placed in the measuring tray of a plate reader (Spectramax M5, Molecular Devices), and an initial UV–vis measurement at 530 nm was performed (Fig. 2d and Extended Data Fig. 2). The 96-well plate was irradiated with light at 530 nm. The sample was irradiated at intensities of either 0.76 W cm^−2^ or 1.49 W cm^−2^. UV–vis measurements were taken at 530 nm every 10 min over 160 min. In the case of non-stimulated nanoreactors, absorbance measurements were performed as kinetics at 530 nm every 10 min for 160 min.

### Evaluation of urea hydrolysis by PISA–urease nanoreactors

A 1-μl volume of a PISA–urease dispersion was introduced into 295 μl of an aqueous solution of MR (7.4 × 10^−1^ mM), urea (8.3 × 10^−1^ M) and saturated ethyl acetate (pH 7.8) contained in a Corning 96-well microplate (poly(styrene), non-binding). The plate was placed in the measuring tray of a plate reader (Spectramax M5, Molecular Devices) and kinetics measurements were taken at 530 nm every 10 s over 40 min (Fig. 3c).

### Synthesis of PNIPAAm-*co*-PDMAEMA-*co*-PNBA gels

*N*-isopropyl acrylamide (NIPAAm) (200 mg, 1.76 mmol), 2-dimethylaminoethyl methacrylate (DMAEMA) (0.57 ml, 538 mg, 3.4 mmol), *N*,*N*′-methylenebisacrylamide (9.75 mg, 6.32 × 10^−2^ mmol), Nile blue acrylamide (1 mg, 2.45 µmol), lithium phenyl-2,4,6-trimethylbenzoylphosphinate (LAP; 2 mg, 6.80 μmol) and PEG *M*_n_ ≈ 2,000 g mol^−1^ were dissolved in 250 μl of ultrapure water. A 60-μl volume of this solution was deposited between two polydimethylsiloxane discs (diameter, 2 cm) and placed under a UV lamp at 365 nm for 10 min. The discs were then separated and immersed in a water bath overnight to promote the separation of the gel from the disc and the elution of impurities. Smaller gels were cut out with a 2-mm biopsy punch to be employed for light-mediated swelling and deswelling experiments (Fig. 5 and Supplementary Fig. 20).

## Online content

Any methods, additional references, Nature Research reporting summaries, source data, extended data, supplementary information, acknowledgements, peer review information; details of author contributions and competing interests; and statements of data and code availability are available at 10.1038/s41557-022-01062-4.

## Supplementary information

Supplementary Information Supplementary sections 1–17, Figs. 1–21 and references.

## Data Availability

Data supporting the findings of this study are contained within the manuscript and its Supplementary Information files, and raw research data are available online at 10.5281/zenodo.6795274. Source data are provided with this Paper.

## Source data

Source Data Fig. 2 Absorbance data, statistical source data.
Source Data Fig. 3 Absorbance data, statistical source data.
Source Data Fig. 4 Absorbance data, statistical source data.
Source Data Fig. 5 Statistical source data.
Source Data Extended Data Fig. 2 Absorbance data, statistical source data.
Source Data Extended Data Fig. 3 Absorbance data, statistical source data.
Source Data Extended Data Fig. 4 Absorbance data, statistical source data.
Source Data Extended Data Fig. 5 Absorbance data, statistical source data.
Source Data Extended Data Fig. 6 Absorbance data, statistical source data.
Source Data Extended Data Fig. 7 Absorbance data, statistical source data.
Source Data Extended Data Fig. 8 Absorbance data, statistical source data.
Source Data Extended Data Fig. 9 Absorbance data, statistical source data.
Source Data Extended Data Fig. 10 Absorbance data, statistical source data.

## References

[1] Merindol R, Walther A (2017). Materials learning from life: concepts for active, adaptive and autonomous molecular systems. Chem. Soc. Rev..

[2] Marguet M, Bonduelle C, Lecommandoux S (2013). Multicompartmentalized polymeric systems: towards biomimetic cellular structure and function. Chem. Soc. Rev..

[3] Küchler A, Yoshimoto M, Luginbühl S, Mavelli F, Walde P (2016). Enzymatic reactions in confined environments. Nat. Nanotechnol..

[4] Palivan CG (2016). Bioinspired polymer vesicles and membranes for biological and medical applications. Chem. Soc. Rev..

[5] Discher DE, Eisenberg A (2002). Polymer vesicles. Science.

[6] Che H, Cao S, van Hest JC (2018). Feedback-induced temporal control of ‘breathing’ polymersomes to create self-adaptive nanoreactors. J. Am. Chem. Soc..

[7] Rifaie-Graham O (2018). Wavelength-selective light-responsive DASA-functionalized polymersome nanoreactors. J. Am. Chem. Soc..

[8] Rifaie-Graham O (2021). Shear stress-responsive polymersome nanoreactors inspired by the marine bioluminescence of dinoflagellates. Angew. Chem. Int. Ed..

[9] Moreno S (2020). Light-driven proton transfer for cyclic and temporal switching of enzymatic nanoreactors. Small.

[10] Wang X (2021). Feedback-induced and oscillating pH regulation of a binary enzyme-polymersomes system. Chem. Mater..

[11] Wang L, Li Q (2018). Photochromism into nanosystems: towards lighting up the future nanoworld. Chem. Soc. Rev..

[12] Jochum FD, Théato P (2013). Temperature- and light-responsive smart polymer materials. Chem. Soc. Rev..

[13] Molla MR (2018). Dynamic actuation of glassy polymersomes through isomerization of a single azobenzene unit at the block copolymer interface. Nat. Chem..

[14] Wang X (2015). Reversibly switching bilayer permeability and release modules of photochromic polymersomes stabilized by cooperative noncovalent interactions. J. Am. Chem. Soc..

[15] Jerca FA, Jerca VV, Hoogenboom R (2022). Advances and opportunities in the exciting world of azobenzenes. Nat. Rev. Chem..

[16] Helmy S (2014). Photoswitching using visible light: a new class of organic photochromic molecules. J. Am. Chem. Soc..

[17] Hemmer JR (2018). Controlling dark equilibria and enhancing donor-acceptor Stenhouse adduct photoswitching properties through carbon acid design. J. Am. Chem. Soc..

[18] Hemmer JR (2016). Tunable visible and near infrared photoswitches. J. Am. Chem. Soc..

[19] Mallo N (2016). Photochromic switching behaviour of donor-acceptor Stenhouse adducts in organic solvents. Chem. Commun..

[20] Lerch MM, Wezenberg SJ, Szymanski W, Feringa BL (2016). Unraveling the photoswitching mechanism in donor-acceptor Stenhouse adducts. J. Am. Chem. Soc..

[21] Diaz YJ (2017). A versatile and highly selective colorimetric sensor for the detection of amines. Chem. Eur. J..

[22] Dolinski ND (2017). A versatile approach for in situ monitoring of photoswitches and photopolymerizations. ChemPhotoChem.

[23] Jia S (2017). Investigation of donor-acceptor Stenhouse adducts as new visible wavelength-responsive switching elements for lipid-based liquid crystalline systems. Langmuir.

[24] Ulrich S (2017). Visible light-responsive DASA-polymer conjugates. ACS Macro Lett..

[25] Yap JE, Zhang L, Lovegrove JT, Beves JE, Stenzel MH (2020). Visible light-responsive drug delivery nanoparticle via donor-acceptor Stenhouse adducts (DASA). Macromol. Rapid Commun..

[26] Senthilkumar T (2018). Conjugated polymer nanoparticles with appended photo‐responsive units for controlled drug delivery, release and imaging. Angew. Chem. Int. Ed..

[27] Ulrich S (2021). Nano-3D-printed photochromic micro-objects. Small.

[28] Lee J (2020). Tunable photothermal actuation enabled by photoswitching of donor-acceptor Stenhouse adducts. ACS Appl. Mater. Interfaces.

[29] Chen Q (2019). Stable activated furan and donor–acceptor Stenhouse adduct polymer conjugates as chemical and thermal sensors. Macromolecules.

[30] Blackman LD (2017). Permeable protein-loaded polymersome cascade nanoreactors by polymerization-induced self-assembly. ACS Macro Lett..

[31] Hu G, Pojman JA, Scott SK, Wrobel MM, Taylor AF (2010). Base-catalyzed feedback in the urea-urease reaction. J. Phys. Chem. B.

[32] Heuser T, Merindol R, Loescher S, Klaus A, Walther A (2017). Photonic devices out of equilibrium: transient memory, signal propagation and sensing. Adv. Mater..

[33] Jee E, Bánsági T, Taylor AF, Pojman JA (2016). Temporal control of gelation and polymerization fronts driven by an autocatalytic enzyme reaction. Angew. Chem. Int. Ed..

[34] Che H, Buddingh’ BC, van Hest JC (2017). Self‐regulated and temporal control of a ‘breathing’ microgel mediated by enzymatic reaction. Angew. Chem. Int. Ed..

[35] Gao N (2021). Chemical-mediated translocation in protocell-based microactuators. Nat. Chem.

[36] Wrobel MM (2012). pH wave-front propagation in the urea-urease reaction. Biophys. J..

[37] Li, Y. & Ju, D. in *Neurotoxicity of Nanomaterials and Nanomedicine* (eds Jiang, X. & Gao, H.) 285–329 (Elsevier, 2017).

[38] Grzybowski BA, Whitesides GM (2002). Dynamic aggregation of chiral spinners. Science.

[39] Kundu PK (2015). Light-controlled self-assembly of non-photoresponsive nanoparticles. Nat. Chem..

[40] Iamsaard S (2014). Conversion of light into macroscopic helical motion. Nat. Chem..

[41] Zhao H (2016). Reversible trapping and reaction acceleration within dynamically self-assembling nanoflasks. Nat. Nanotechnol..

[42] Leira-Iglesias J, Tassoni A, Adachi T, Stich M, Hermans TM (2018). Oscillations, travelling fronts and patterns in a supramolecular system. Nat. Nanotechnol..

[43] Monreal Santiago G, Liu K, Browne WR, Otto S (2020). Emergence of light-driven protometabolism on recruitment of a photocatalytic cofactor by a self-replicator. Nat. Chem..

[44] Semenov SN (2015). Rational design of functional and tunable oscillating enzymatic networks. Nat. Chem..

[45] van der Helm MP, de Beun T, Eelkema R (2021). On the use of catalysis to bias reaction pathways in out-of-equilibrium systems. Chem. Sci..

[46] Boekhoven J, Hendriksen WE, Koper GJ, Eelkema R, van Esch JH (2015). Transient assembly of active materials fueled by a chemical reaction. Science.

[47] Maiti S, Fortunati I, Ferrante C, Scrimin P, Prins LJ (2016). Dissipative self-assembly of vesicular nanoreactors. Nat. Chem..

[48] Rodríguez-Arco L, Li M, Mann S (2017). Phagocytosis-inspired behaviour in synthetic protocell communities of compartmentalized colloidal objects. Nat. Mater..

[49] Pearce S, Perez-Mercader J (2021). Chemoadaptive polymeric assemblies by integrated chemical feedback in self-assembled synthetic protocells. ACS Cent. Sci..

[50] Kapusta, P. *Absolute Diffusion Coefficients: Compilation of Reference Data for FCS Calibration* (PicoQuant, 2010); https://www.picoquant.com/images/uploads/page/files/7353/appnote_diffusioncoefficients.pdf

[51] Müller P, Schwille P, Weidemann T (2014). PyCorrFit—generic data evaluation for fluorescence correlation spectroscopy. Bioinformatics.

[52] Eberhardt M, Mruk R, Zentel R, Théato P (2005). Synthesis of pentafluorophenyl(meth)acrylate polymers: new precursor polymers for the synthesis of multifunctional materials. Eur. Polym. J..

[53] Perrier S, Takolpuckdee P, Mars CA (2005). Reversible addition-fragmentation chain transfer polymerization: end group modification for functionalized polymers and chain transfer agent recovery. Macromolecules.

[54] Yin H (2012). Solvent-free copper-catalyzed N-arylation of amino alcohols and diamines with aryl halides. Tetrahedron Lett..

[55] Helmy S, Oh S, Leibfarth FA, Hawker CJ, Read de Alaniz J (2014). Design and synthesis of donor-acceptor Stenhouse adducts: a visible light photoswitch derived from furfural. J. Org. Chem.

