# Supplementary material for: Photoswitchable gating of non-equilibrium enzymatic feedback in chemically communicating polymersome nanoreactors
Source: Nat Chem. 2022 Nov 7;15(1):110–8. doi: 10.1038/s41557-022-01062-4 (PMC9836937; doi:10.1038/s41557-022-01062-4)
Supplement: Supplementary file 1 — Supplementary sections 1–17, Figs. 1–21 and references. [file 41557_2022_1062_MOESM1_ESM.pdf]

nature chemistry

Article https://doi.org/10.1038/s41557-022-01062-4

Photoswitchable gating of non-equilibrium enzymatic feedback in chemically communicating polymersome nanoreactors

In the format provided by the authors and unedited

Supplementary Information

Photoswitchable gating of non-equilibrium enzymatic feedback in chemically communicating polymersome nanoreactors

Omar Rifaie-Graham[1], Jonathan Yeow[1], Adrian Najer[1], Richard Wang[1], Rujie Sun[1], Kun Zhou[1], Tristan N. Dell[1], Christopher Adrianus[1], Chalaisorn Thanapongpibul[1], Mohamed Chami[2], Stephen Mann[3,4,5], Javier Read de Alaniz[6], Molly M. Stevens[1*]

[1]Department of Materials, Department of Bioengineering, and Institute of Biomedical Engineering at Imperial College London, Prince Consort Rd, SW7 2AZ London, South Kensington, UK

[2]BioEM lab, Biozentrum, University of Basel, Mattenstrasse 26, 4056, Basel, Switzerland

[3]Centre for Protolife Research and Centre for Organized Matter Chemistry, School of Chemistry, University of Bristol, Bristol BS8 1TS, UK

[4]School of Materials Science and Engineering, Shanghai Jiao Tong University, Shanghai, 200240, P. R. China.

[5]Max Planck-Bristol Centre for Minimal Biology, School of Chemistry, University of Bristol, Bristol BS8 1TS, United Kingdom.

[6]Department of Chemistry and Biochemistry, University of California, Santa Barbara, California 93106, United States

Corresponding author: m.stevens@imperial.ac.uk

Supplementary Information 1. Evaluation of enzymatic activity of free esterase and urease in unbuffered conditions

A series of experiments was performed to evaluate the catalytic activity of the enzymes throughout a range of pH values. To showcase the catalytic activity of urease in acidic conditions, an aqueous solution of methyl red ($7.4\cdot10^{-1}$ µmol·mL^{-1}), urea ($8.3\cdot10^{-1}$ mmol·mL^{-1}), and saturated ethyl acetate was prepared and acidified with acetic acid ($1.7\cdot10^{-1}$ mmol) to pH = 6.4. 300 µL of this solution were transferred to a well of a Corning 96-well microplate (poly(styrene), non-binding). A pH electrode was immersed in the solution followed by spiking with urease. The final concentration of urease was 12 pmol·mL^{-1}. The pH evolution was monitored over time until a plateau was reached at pH ~ 8.5 (Supplementary Figure 1). To test the activity of the esterase in basic conditions, the solution was further spiked with esterase. The final concentration of esterase was 2 nmol·mL^{-1}. Then, the pH was monitored over time. The pH values rapidly evolved to become acidic, achieving a lower plateau at pH ~ 4.5. To further test the activity of urease in such an acidic environment, the solution was finally spiked with urease. The final concentration of urease in the solution was 73 pmol·mL^{-1}. The pH was again monitored over time. An increase of pH was observed, reaching a plateau at pH ~ 6.5.

Literature reports that urease is self-inhibited at alkaline pH ~ 9.[1] Thus, the initial plateau formation at alkaline pH is expected. Esterase from pig liver presents an optimal pH ~ 8.[2] By introducing excess of esterase to the reaction, the formation of acid overcame the production of base by urease which was far from its optimal pH (pH ~ 6).[1-2] The esterase becomes practically self-inhibited at pH < 4.5, explaining the formation of a lower plateau in the evolution of pH.[2] Therefore, the addition of urease allowed the increase in pH. The reaction eventually stabilised its pH values probably due to the simultaneous formation of acid and base by the antagonistic enzymes.

Supplementary Information 2. General synthetic procedure of amphiphilic block copolymers

The DASA amphiphilic block copolymer was synthesised based on poly(ethylene glycol) (PEG) as a hydrophilic stabilising block and poly(butyl acrylate) (PBA) as the hydrophobic core forming polymer. For this purpose, a poly(ethylene glycol) (PEG) chain-transfer agent was chain-extended by reversible addition-fragmentation chain transfer (RAFT) co-polymerisation of butyl acrylate (BA) and pentafluorophenyl acrylate (PFPA) to yield PEG_{43}-*b*-(PBA_{30}-*co*-$PPFPA_{3}$). The polymer was end-modified with excess AIBN to substitute the dithioester end group with a 2-cyanoisopropyl group to avoid the formation of nucleophiles during the subsequent amidation reaction. Then, the activated pentafluorophenyl ester was modified with N-(4-methoxyphenyl)-1,3-diaminepropane (MPDP), to form the aromatic amine DASA precursor polymer. Finally, the aromatic amines were modified with a Meldrum's acid-based furan adduct to yield the DASA block copolymer (polymer synthesis experimental and data: Supplementary Information 3-5 and Supplementary Figures 1-5).

Permeable nanoreactors were synthesised by photoinitiated polymerisation induced self-assembly (photo-PISA) in the presence of urease (Supplementary Figures 1 and 2). This was achieved by chain-extension of a poly(ethylene glycol) macrochain-transfer agent and with the monomer 2-hydroxypropyl methacrylate in the presence of urease employing blue light (λ = 405 nm), yielding PISA-urease.

Supplementary Information 3. Characterisation of PEG-*b*-(PBA-*co*-PPFPA) and calculation of number average molecular weight (M_n) of the hydrophilic block

According to GPC analysis, the polymer presented a Đ = 1.29 and a poly(methyl methacrylate)-apparent M_n of 10300 g mol^{-1} (Supplementary Figure 2). The ^1H NMR spectrum of this polymer (Supplementary Figure 5A) allowed the determination of the M_n of the poly(ethylene glycol) block by employing the following formula:

$$PEG\ M_n(g\ mol^{-1}) = \frac{3e}{4f} \cdot 44.05 + M$$

Where *M* corresponded to the molar mass of the methoxy end-group of the PEG block, 31.03 g mol^{-1}. The number of ethylene glycol repeat units was estimated to be 43.5 $chain^{-1}$ and the M_n of the PEG block was determined to be 1947 g mol^{-1}.

Supplementary Information 4. Synthesis of aromatic amine DASA precursor diblock copolymer

The degree of functionalisation of the hydrophobic block was calculated from the integrals of the ^1H NMR spectrum by employing the following equation (peak assignment and integrals in Supplementary Figure 5B):

$$amide\ content\ (\ mol\ \%) = \frac{e + f}{e + f + 2a} \cdot 100$$

9.9 mol% of the hydrophobic residues were functionalised and 90.1 mol% corresponded to poly(butyl acrylate) units. Given that the M_n of the PEG block (calculated by ^1H NMR, Supplementary Figure 5A) was 1947 g mol^{-1}, the M_n of the hydrophobic block was calculated from the ratio of the repeating units in the hydrophobic block:

$$\frac{hydrophobic\ block\ repeating\ units}{hydrophylic\ block\ repeating\ units} = \frac{2a + e + f}{i}$$

The number of repeat units in the hydrophobic block was estimated to be 34 $chain^{-1}$. By the calculation of the molar ratio and the molecular mass of the repeat units, it was estimated that the M_n of the hydrophobic block was 6370 g mol^{-1}, and the M_n of the aromatic amine DASA precursor diblock copolymer was 8320 g mol^{-1}.

Supplementary Information 5. Modification of aromatic amine DASA precursor polymer to yield the DASA polymer

Full DASA functionalisation on the secondary aromatic amine was determined according to ^1H NMR (Supplementary Figure 5D), by comparison of the aromatic protons of the anisole ring and the first triene-enol proton adjacent to the Meldrum's acid according to the following equation:

$$Degree\ of\ functionalisation\ (\%) = \frac{4c}{a + b} \cdot 100$$

Assuming that full functionalisation had been generated, the M_n of the DASA polymer could be calculated:

$$M_n DASA\ polymer\ (g\ mol^{-1}) = M_n Aromatic\ amine\ polymer + \frac{H \cdot A \cdot Z}{100}$$

Where *H* is the number of hydrophobic residues, 34, *A* is the amide mol% in the hydrophobic block, 9.9 %, and *Z* is the molar mass of the Meldrum's acid-based furan adduct, 222.2 g mol^{-1}. Therefore, the M_n of the DASA polymer was calculated to be 9070 g mol^{-1}.

Supplementary Information 6. Characterisation of DASA-esterase nanoreactors

Evaluation of trypsinisation effect on DASA-esterase nanoreactors. We employed trypsin to digest the non-encapsulated enzyme as described in the methods section of the main manuscript. To evaluate that the catalytic activity was restricted to the encapsulated enzyme, a series of control experiments was performed. For this purpose, 5 µL of trypsinised esterase solution at self-assembly concentration was introduced to 295 µL of an aqueous solution of methyl red ($7.4 \cdot 10^{-1}$ µmol·mL^{-1}) and saturated ethyl acetate (pH = 7.8) and kinetic absorbance measurements over 3 h were performed as explained in the methods section of the main manuscript (Supplementary Figure 8A). As a comparison, DASA-esterase nanoreactors which had been subjected to trypsinisation after the self-assembly process were subjected to the same reaction conditions in the presence of green light at 1.49 mW·cm^{-2} and in darkness. Changes in absorbance (MR), and hence pH, over time were only observed in the irradiated DASA-esterase sample. Such results indicated that the catalytic activity of non-encapsulated esterase had been successfully quenched by trypsin-mediated digestion and confirmed trypsinisation as an effective method to restrict the catalysis to the compartmentalised enzyme. The digestion of esterase was further investigated by fluorescence correlation spectroscopy (FCS) which allows to perform diffusion-based measurements of fluorescent species in liquid solutions and dispersions. Thus, solutions of rhodamine B-labelled esterase (RhB-esterase) were incubated with trypsin and compared to free sulforhodamine B (SRB) and non-trypsinised RhB-esterase. The normalised FCS autocorrelation curves showed slower diffusion times (τ_D) for RhB-esterase compared to free SRB confirming successful labelling and purification of the enzyme (Supplementary Figure 8B). When RhB-esterase was treated with trypsin, the normalised FCS autocorrelation curve revealed a characteristic τ_D between non-digested RhB-esterase and the free dye. The resulting molecular hydrodynamic diameters (D_H) were 1.2 nm (SD = 0.1 nm) for SRB, 2.0 nm (SD = 0.4 nm) for trypsinised RhB-esterase, and 4.1 nm (SD = 0.1 nm) for RhB-esterase (Supplementary Figure 8C) confirming the hydrolysis of the esterase by trypsin. We sought to calculate the retained enzymatic activity of the DASA-esterase nanoreactors. Thus, we treated 200 µL of the trypsinised DASA-esterase nanoreactors with 40 µL of an aqueous solution containing the trypsin inhibitor aprotinin (30.7 nmol) and incubated the sample at 37 °C overnight. The nanoreactors were then lysed in 20 mmol·mL^{-1} of Triton X-100 to a final volume of 1 mL for 10 min according to a previously reported protocol.[3] Enzymatic activity was tested by transferring 50 µL of the treated solution to a 96 well-microplate containing 250 µL of MR ($7.4 \cdot 10^{-1}$ µmol·mL^{-1}) and saturated ethyl acetate (pH = 8.5) followed by monitoring of absorbance over time at λ = 530 nm. As a control, a fresh non-encapsulated esterase aliquot at equal concentration was treated by solvent exchange at equal conditions to the formation of DASA-esterase nanoreactors in absence of the DASA polymer and trypsin, and was further spiked with the trypsin inhibitor and Triton X-100. To conserve enzymatic activity, the sample was not incubated at 37 °C and was immediately tested against ethyl acetate hydrolysis in the presence of MR at equal concentrations to the dissolved DASA-esterase nanoreactor sample. The retention of enzymatic activity was calculated by employing the following formula:

$$Enzymatic\ activity\ retention = \frac{m_{DE} - m_{blank}}{m_{FE} - m_{blank}}$$

Where, m_{DE} is the slope of the reaction catalysed by the enzyme liberated from the DASA-esterase nanoreactors, m_{blank} is the slope of the reaction in absence of esterase, and m_{FE} is the slope catalysed by the treated free esterase. It was calculated that an equivalent of 12.8 % of the enzyme activity was retained, which corresponds to the encapsulation efficiency (assuming no loss of enzyme activity for the loading and trypsinisation process for DASA-esterase). This data also confirms successful protection of esterase from proteases when encapsulated within DASA polymersomes. To cross-validate loading efficiency, DASA polymersomes were also loaded with Alexa Fluor 647-

esterase and measured by FCS after trypsinisation. Loading had to be performed at lower enzyme concentrations than normal (525 nM esterase-AF647, 88 µg·mL^{-1}) due to the inability to purify away unencapsulated enzyme fragments. The FCS autocorrelation curves showed slower τ_D for Alexa Fluor 647-esterase compared to free Alexa Fluor 647, confirming successful labelling and purification of the enzyme (Supplementary Figure 9). Moreover, Alexa Fluor 647-esterase-loaded nanoreactors showed a particle fraction of 7.5 % (two-component fits due to measurement of non-purified samples, orange curve), which corresponds to the encapsulation efficiency in this experimental setup without purification. The control mixture of free Alexa Fluor 647-esterase with unloaded DASA polymersomes did not reveal a significant particle fraction when applying a two-component fit (blue curve).

Finally, FCS was also employed to directly measure the DASA photoswitching, whilst simultaneously obtaining the nanoparticle hydrodynamic diameter (D_H) (Supplementary Figure 10).

Influence of photo-induced isomerisation of DASA on particle size. Various reports have shown that the permeabilization of stimuli-responsive polymersome membranes to small molecules can be induced by a swelling mechanism.[4-5] DASAs isomerise from a colourful, fluorescent, non-polar triene-enol to a colourless, non-fluorescent, and more polar cyclopentenone isomer. Thus, we investigated if the photoswitching of DASAs within the polymersome membrane induces size changes through FCS. For this purpose, the fluorescence emission of the DASA polymersomes was recorded whilst exciting at 561 nm. As expected, during irradiation with the 561 nm laser beam, the fluorescence intensity (count rate) decreased over time indicating the fast (< 60 s) photoisomerization of DASA during the FCS measurement (Supplementary Figure 10A). Interestingly, the obtained D_H did not change over time and across 6 technical replicates (Supplementary Figure 10B). To mimic the light irradiation performed in all the other experiments, we also performed FCS measurements after offline irradiation of the samples for 10 min, 30 min, and 60 min with the LED array (green light, 530 nm, 4.31 mW·cm^{-2}), alongside a control of 60 min in darkness after 60 min of LED irradiation (Supplementary Figure 10C). The results showed no apparent change in D_H, demonstrating that the change in semi-permeability of the DASA polymersomes with light irradiation is not associated to a swelling process. In addition, similar results have been observed in spiropyran-functionalised polymersomes[6] and DASA polymersomes composed of poly(ethylene glycol)-*b*-poly(hexyl methacrylate).[7]

Supplementary Information 7. Calculation of urease protein units per PISA-urease nanoreactor

FCS measurements were employed to evaluate the encapsulation of urease in PISA polymersomes. Therefore, rhodamine B-labelled urease (RhB-urease) was encapsulated in PISA-urease nanoreactors and compared to free RhB-urease. The FCS data revealed different D_H for free and loaded RhB-urease indicating successful encapsulation and purification of the PISA nanoreactors (Supplementary Figures 11A and 11B). Moreover, an average of 31 (SD = 9) urease enzymes per PISA-urease nanoreactor unit was achieved (Supplementary Figure 11C).

Supplementary Information 8. Evaluation of photoinduced negative feedback-loop by addition of free esterase

To prove that the interruption of the catalytic reaction was not due to the total consumption of chemical fuel, the catalytic activity of a DASA-esterase dispersion in the presence of methyl red and ethyl acetate was probed by absorbance measurements as described in the methods section of the main manuscript. The samples were irradiated with green light (λ = 530 nm) at 1.49 mW·cm^{-2} for 160 min (Figure 2F). After formation of the plateau of absorbance values, 5 µL of an aqueous solution

containing 60 pmol of free esterase was added. An abrupt increase in absorbance was observed over time. This process indicated that the formation of the plateau was not due to the total consumption of ethyl acetate but was due to the formation of a photoinduced negative feedback-loop by accumulation of the protonated methyl red photomask. pH values were calculated by fitting the absorbance values to the pH vs Absorbance calibration curve in Supplementary Figure 21 (Supplementary Figure 15).

Supplementary Information 9. Evaluation of photoinduced negative feedback-loop in absence of a photomask.

To prove that the photoinduced inhibition of DASA-esterase catalytic activity was produced by the photomasking effect of accumulated MRH we sought to catalyse acetic acid formation in the presence of bromocresol purple (BP) instead of MR. BP transitions from a non-protonated state with λ_{max} = 585 nm to a protonated state (BPH) λ_{max} = 430 nm at similar pH values to MR^- (Extended Data Figure 2A). 2 µL and 10 µL of 100 times diluted dispersion of the DASA-esterase nanoreactors were added to aqueous solutions of saturated ethyl acetate and BP (0.12 $\mu mol \cdot mL^{-1}$), and irradiated with green light (λ= 530 nm) at 0.76 $mW \cdot cm^{-2}$ (Extended Data Figure 2B) and followed the protonation process by kinetic UV-Vis measurements at λ = 430 nm. When DASA-esterase nanoreactors at the lower concentrations were employed, plateau formation was not observed over time. Instead, plateau formation was observed with higher concentrations of the DASA-esterase nanoreactors. As control, a reaction in the presence of free esterase instead of the DASA-esterase nanoreactors was performed. The reaction reached plateau at similar absorbance values to the reaction catalysed by higher concentrations of the DASA-esterase nanoreactors. The results indicated that the plateau formation was not an inhibition of the catalytic process. Instead, the absorbance values reached plateau indicating that the dye was fully protonated and did not interrupt the catalytic activity of the DASA-esterase nanoreactors. In comparison, when DASA-esterase nanoreactors catalysed the transformation of ethyl acetate to acetic acid in the presence of MR at the same concentrations as the reactions in the presence of BP, plateau formation was observed (Extended Data Figure 2). However, at higher DASA-esterase nanoreactor concentrations the reaction was not fully interrupted over the same period. We hypothesise that in these conditions, the concentration of accumulated MRH was not sufficient to quench the catalytic activity of the DASA-esterase nanoreactors. Compared to experiments in the presence of BP, the results indicated that the accumulation of MRH acted as a photomask and was responsible for the inhibition of the catalytic activity of DASA-esterase producing an effective negative feedback loop.

Supplementary Information 10. Enzyme stability to green light irradiation $\lambda_{530\ nm}$.

Stability of porcine liver esterase to green light irradiation. A solution of esterase (5.9 $nmol \cdot mL^{-1}$) was irradiated at either 1.49 $mW \cdot cm^{-2}$ for 160 min or 4.31 $mW \cdot cm^{-2}$ for 80 min (Supplementary Figure 16A and 19A). 5 µL of each solution was added to 295 µL of a solution of MR ($7.4 \cdot 10^{-1}$ $\mu mol \cdot mL^{-1}$) and saturated ethyl acetate (pH = 8.5). The activity of the enzymes was measured by kinetic UV-Vis spectroscopy at λ = 530 nm. As control, the activity of the non-irradiated enzyme was performed in equal conditions.

Stability of Jack Bean urease to green light irradiation. A solution of urease (2.7 $nmol \cdot mL^{-1}$) was irradiated at either 1.49 $mW \cdot cm^{-2}$ for 160 min or 4.31 $mW \cdot cm^{-2}$ for 80 min (Supplementary Figure 16B and 19B). 5 µL of each solution was added to 295 µL of a solution of MR ($7.4 \cdot 10^{-1}$ $\mu mol \cdot mL^{-1}$) and urea (0.8 $mmol \cdot mL^{-1}$) (pH = 7.4). The activity of the enzymes was measured by kinetic UV-Vis spectroscopy at λ = 530 nm. As control, the activity of the non-irradiated enzyme was performed in equal conditions.

Observable differences between the irradiated and non-irradiated enzymes were not observed, confirming their stability in the presence of green light.

Supplementary Information 11. Evaluation of pH-mediated negative feedback-loop by addition of free urease and re-initiation by acidification

A series of experiments was performed to probe that the inhibition of the catalytic activity of PISA-urease nanoreactors was a pH mediated effect. Therefore, urea was hydrolysed by PISA-urease and absorbance measurements corresponding to the deprotonation of methyl red were performed as explained in the methods section of the main manuscript. After the formation of a lower absorbance plateau, 10 µL of an aqueous solution containing $1.7 \cdot 10^{-1}$ nmol of free urease was added and the absorbance was followed over time (Figure 3C). The absorbance values did not evolve during this time period. The results indicated that urease was inhibited due to the alkalinisation of the medium. To confirm such observations, aqueous dispersions containing PISA-urease nanoreactors, urea, ethyl acetate, and methyl red as described in the methods section of the main manuscript were acidified after lower absorbance plateau formation by addition of 10 µL of an aqueous solution containing 24 nmol of acetic acid. After the increase in absorbance by protonation of methyl red, the absorbance values rapidly decayed and formed a plateau at similar pH values to the previous cycle. First, this phenomenon indicated that the catalytic activity of PISA-urease was not limited by the total consumption of urea as substrate. Moreover, the catalytic activity of PISA-urease was activated at acidic pH and was inhibited at alkaline pH (~8.6). The results confirmed that a pH-mediated negative feedback-loop had been generated for PISA-urease nanoreactors. pH values were calculated by fitting the absorbance values to the pH vs absorbance calibration curve in Supplementary Information 12 (Figure 3D).

Supplementary Information 12. Correlation of absorbance and pH in solutions containing methyl red

Aliquots of 300 µL of an aqueous solution of methyl red ($7.4 \cdot 10^{-1}$ µmol·mL^{-1}), urea ($8.3 \cdot 10^{-1}$ mmol·mL^{-1}), and saturated ethyl acetate (pH = 7.8) were distributed into wells of a Corning 96-well microplate (poly(styrene), non-binding). The solutions were spiked with sub-microlitre solutions of either acetic acid or NaOH to generate a spectrum of absorbances and pH values. The pH of each well was quantified with a pH electrode and the absorbances were recorded by UV-Vis spectroscopy with a plate reader (Spectramax M5, Molecular Devices) (Supplementary Figure 21). The data was fitted to an exponential decay curve ($r^2 = 0.99$) and the following correlation function was obtained:

$$pH = 6.29479 + 0.7759 \cdot e^{\left(\frac{-(A-0.64238)}{0.16596}\right)} + 0.84582 \cdot e^{\left(\frac{-(A-0.64238)}{1.07726}\right)}$$

Where, *A* corresponds to the absorbance values.

Supplementary Information 13. Light-induced modulation of out-of-equilibrium pH

Light intensity-based modulation of pH. 15 µL of DASA-esterase nanoreactors and 1 µL of PISA-urease nanoreactors were added to 300 µL of an aqueous solution of methyl red ($7.4 \cdot 10^{-1}$ µmol·mL^{-1}), urea ($8.3 \cdot 10^{-1}$ mmol·mL^{-1}), and saturated ethyl acetate (pH = 7.8) contained in a Corning 96-well microplate (poly(styrene), non-binding). The plate was placed in the measuring tray of a plater reader (Spectramax M5, Molecular Devices). After an initial measurement at 530 nm, the samples were irradiated at 0.76 mW·cm^{-2} and 1.49 mW·cm^{-2} and measurements were taken at 530 nm every 10 min. When the absorbance values reached a plateau, the samples were allowed to return their initial alkaline pH in darkness and absorbance measurements were acquired as a kinetic

measurement taken every 15 sec. In addition, a control experiment was performed in darkness and absorbance measurements were also acquired every 15 sec (Figure 4B and Extended Data Figure 3).

Stoichiometry-based modulation of pH. In this case, the volumetric ratio of DASA-esterase : PISA-urease nanoreactors was varied from 1:5, 1:10, and 1:15 µL and added to 300 µL of an aqueous solution of methyl red ($7.4\cdot10^{-1}$ µmol·mL^{-1}), urea ($8.3\cdot10^{-1}$ mmol·mL^{-1}), and saturated ethyl acetate (pH = 7.8) contained in a Corning 96-well microplate (poly(styrene), non-binding). Measurements were performed as for the previously described light intensity-based modulation of pH (Figure 4C and Extended Data Figure 4). When the influence of PBS (10 mM phosphate) was probed, all reactants were employed at the same concentrations.

Supplementary Information 14. Nanoreactor stability to variations of pH.

To evaluate if the nanoreactors could conserve structural integrity during the acidification and alkalinisation of the media, we spiked dispersions containing either DASA-esterase or PISA-urease that were initially at pH = 8.2 with an acetic acid solution transitioning to pH = 6. Both dispersions were incubated at room temperature for 10 min, followed by restoration to pH = 8.2 with a NaOH solution. The samples were analysed before and after the procedure by cryo-TEM and DLS (Extended Data Figure 6). The measurements showed no changes in structure indicating that the function of the nanoreactors remained unaltered throughout the oscillations of pH.

Supplementary Information 15. Catalysis mediated by Intermittent irradiation on DASA-esterase

To prove that the catalytic activity of DASA-esterase nanoreactors can be modulated by a photomasking effect, it should be possible to interrupt the catalytic activity in the absence of light. Thus, the modulation of the catalytic activity by alternation of light irradiation and withdrawal was probed. Therefore, the catalytic activity of DASA-esterase nanoreactors was monitored as described in the methods section of the main manuscript. After an initial absorbance measurement at λ = 530 nm, green light (λ = 530 nm) was irradiated at 1.49 mW·cm^{-2} for 10 min and the absorbance in dark conditions was monitored every 5 min throughout 30 min (Extended Data Figure 7). Then, 3 additional irradiation/darkness cycles were monitored consecutively. The rate of absorbance increased strongly through each irradiation cycle and became slower in the absence of light. Therefore, the results indicate that the hydrolysis of ethyl acetate could be activated and deactivated by alternating green light irradiation and darkness. pH values were calculated by fitting the absorbance values to the pH vs absorbance calibration curve in Supplementary Information 12.

Supplementary Information 16. Circadian rhythm-like modulation of chemical environment

In a typical reaction, 15 µL of DASA-esterase nanoreactors and 2 µL of PISA-urease nanoreactors were added to 300 µL of an aqueous solution of methyl red ($7.4\cdot10^{-1}$ µmol·mL^{-1}), urea ($4.2\cdot10^{-1}$ mmol·mL^{-1}), and saturated ethyl acetate (pH = 7.8) contained in a Corning 96-well microplate (poly(styrene), non-binding). The samples were irradiated with green light (λ = 530 nm) at 4.31 mW·cm^{-2} for 10 min and absorbance measurements were taken every 15 sec for 15 min with a plater reader (Spectramax M5, Molecular Devices) (Supplementary Figure 17). The process was repeated over 8 consecutive cycles and measurements were performed in triplicate. pH values were calculated by fitting the absorbance values to the pH vs absorbance calibration curve in Supplementary Information 12 (Figure 4B). In reactions where the influence of trypsin inhibition was tested, 10 uL of a solution containing 7.7 nmol of aprotinin was added (Supplementary Figure 18). In reactions where the concentration of urea was tested, concentrations of $8.3\cdot10^{-1}$ mmol·mL^{-1} and 1.6 mmol·mL^{-1} were also explored (Extended Data Figure 9). Reactions that explored the influence of

ammonium acetate were performed at concentrations of 10 mM and 50 mM (Extended Data Figure 10).

Supplementary Information 17. Light-induced modulation of pH-responsive hydrogel swelling ratio

PNIPAAm-*co*-PDMAEMA gels prepared as described in the methods section of the main manuscript, were introduced into 300 µL of an aqueous solution of methyl red ($7.4 \cdot 10^{-1}$ µmol·mL^{-1}), urea ($8.3 \cdot 10^{-1}$ mmol·mL^{-1}), and saturated ethyl acetate (pH = 7.8) contained in a Corning 96-well microplate (poly(styrene), non-binding). Unless otherwise stated, 1 µL of PISA-urease and 20 µL of DASA-esterase was added, and the samples were irradiated with green light (λ = 530 nm) at 1.49 mW·cm^{-2} for 4 h. The size of the gels was monitored by photographic imaging employing a dermatoscope (Firefly DE300 Polarizing Handheld USB Digital). Photographs were taken every 10 min. In experiments where the gels were allowed to achieve their original de-swelled state, the samples were placed without irradiation of green light for an additional 8 h. To re-initiate the swelling process, 100 µL of an aqueous solution of saturated ethyl acetate and urea ($8.3 \cdot 10^{-1}$ mmol·mL^{-1}) followed by irradiation of green light (λ = 530 nm) at 1.49 mW·cm^{-2} for 4 h and the samples were allowed to de-swell for an additional 8 h.

The diameter of the gels was quantified with ImageJ (win64). For this purpose, the diameter of the bottom of the wells was employed as reference and set to 1000 a.u.. In practice, defects in the contour of the gels which approximated to the diameter were employed as reference points for the measurements. The swelling ratio was defined as:

$$Swelling\ Ratio\ (\%) = \frac{2\pi r^2}{2\pi r_0^2} \cdot 100$$

Where r_0 is the initial diameter of the gel, and r is the diameter of the gel throughout the experiment.

Supplementary Figures

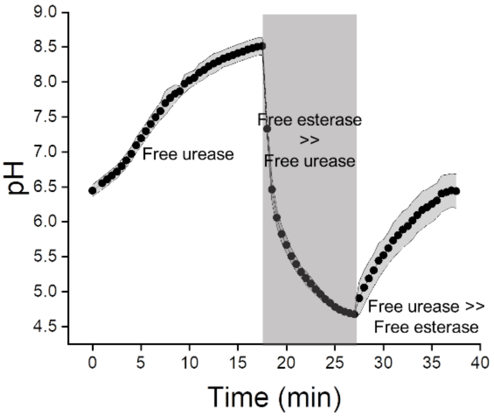

Supplementary Figure 1 │Evolution of pH by alternation of urease and esterase spiking in non-buffered conditions. The solutions were initially spiked with 3.6 pmol of urease, followed by 0.6 pmol esterase, and finally additional 18.3 pmol of urease (*n* = 3 technical replicates, mean ± SD).

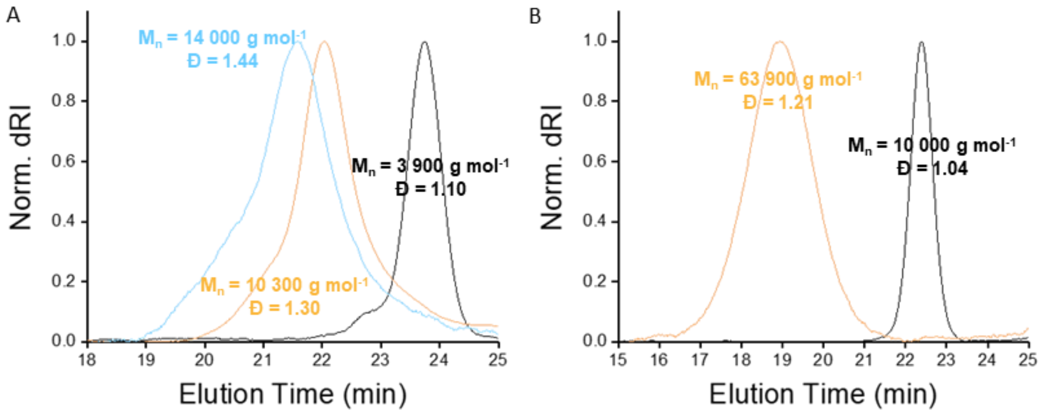

Supplementary Figure 2 |GPC elugrams of synthesised polymers. 0.075 w/w % LiBr in DMF-GPC elugrams of **A**. DASA polymer (blue), PEG-*b*-(PBA-*co*-PPFPA) (orange), PEG-CPADB (black) **B**. PEG-*b*-PHPMA (orange) and PEG-CDTPA (black).

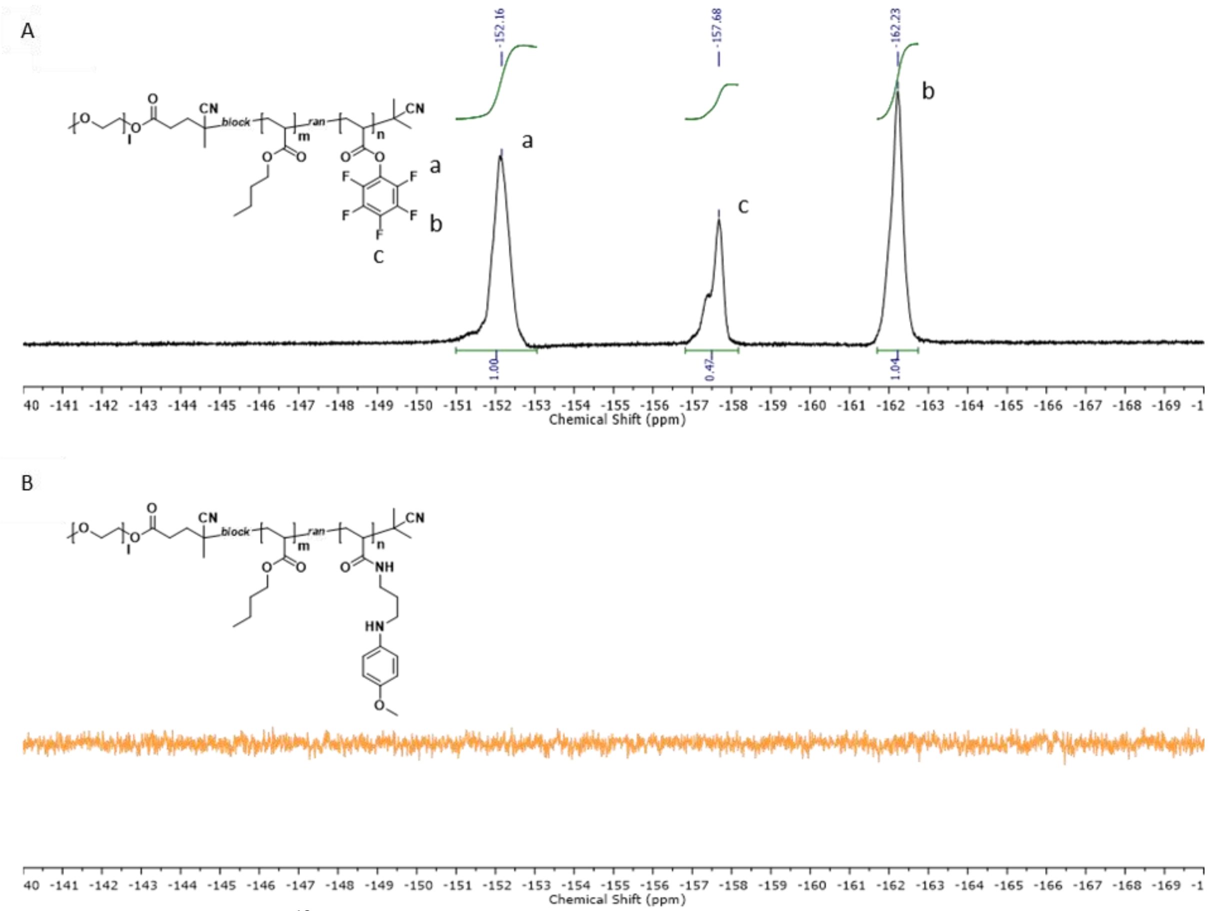

Supplementary Figure 3 |^{19}F NMR of PEG-b-(PBA-*co*-PPFPA) before and after modification with the DASA aromatic amine precursor. A. PEG-*b*-(PBA-*co*-PPFPA) diblock copolymer. **B**. Aromatic amine DASA precursor polymer. The disappearance of the pentafluorophenyl ester fluorine peaks indicate the modification of the polymer with the aromatic amine DASA precursor, which was further confirmed by FT-IR (Supplementary Figure 4).

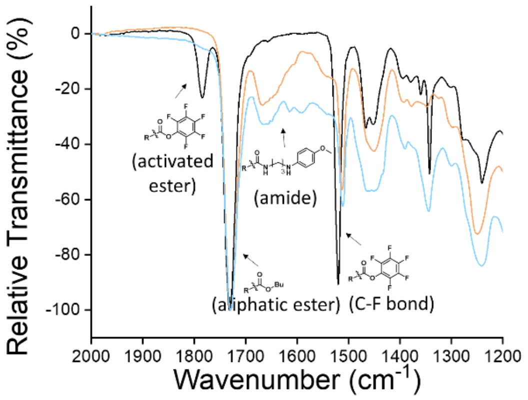

Supplementary Figure 4 | ATR FT-IR of synthetic steps to achieve DASA polymers. The activated ester polymer PEG-*b*-(PBA-*co*-PPFPA) (black) was modified with the aromatic amine precursor MPDP (orange). The disappearance of the activated ester bond coincides with the formation of an amide bond. Reaction with the Meldrum's acid-based furan adduct (blue) generates the DASA polymer.

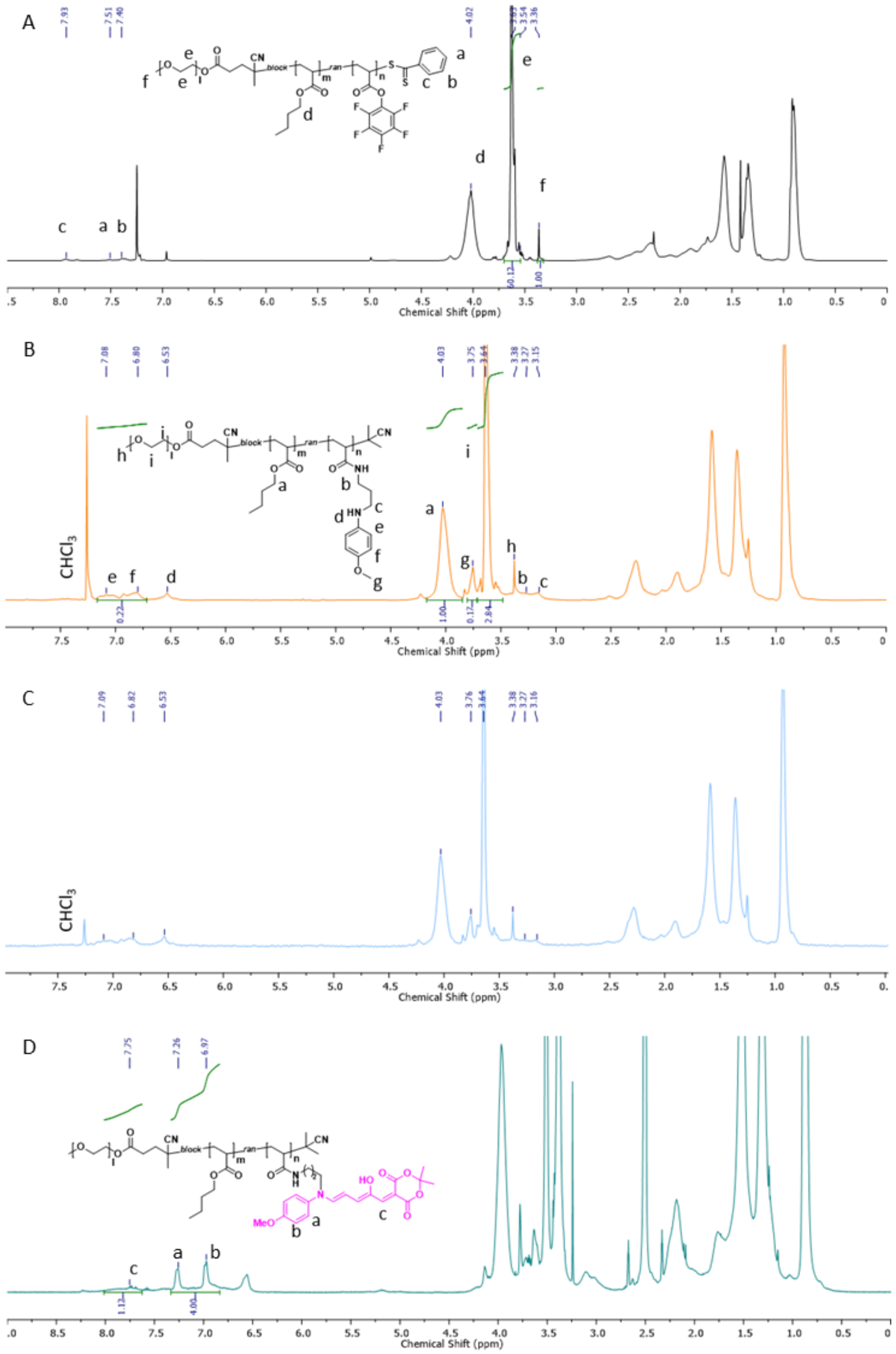

Supplementary Figure 5 │NMR spectroscopy of synthetic steps to achieve DASA polymers. A. ^{1}H NMR of PEG-*b*-(PBA-*co*-PPFPA). **B**. Aromatic amine-modified block copolymer. **C**. Diffusion-edited ^{1}H NMR of the aromatic amine-modified block copolymer. The presence of the highlighted peaks from panel B confirms the functionalisation of the polymer. **D**. ^{1}H NMR of the DASA block copolymer.

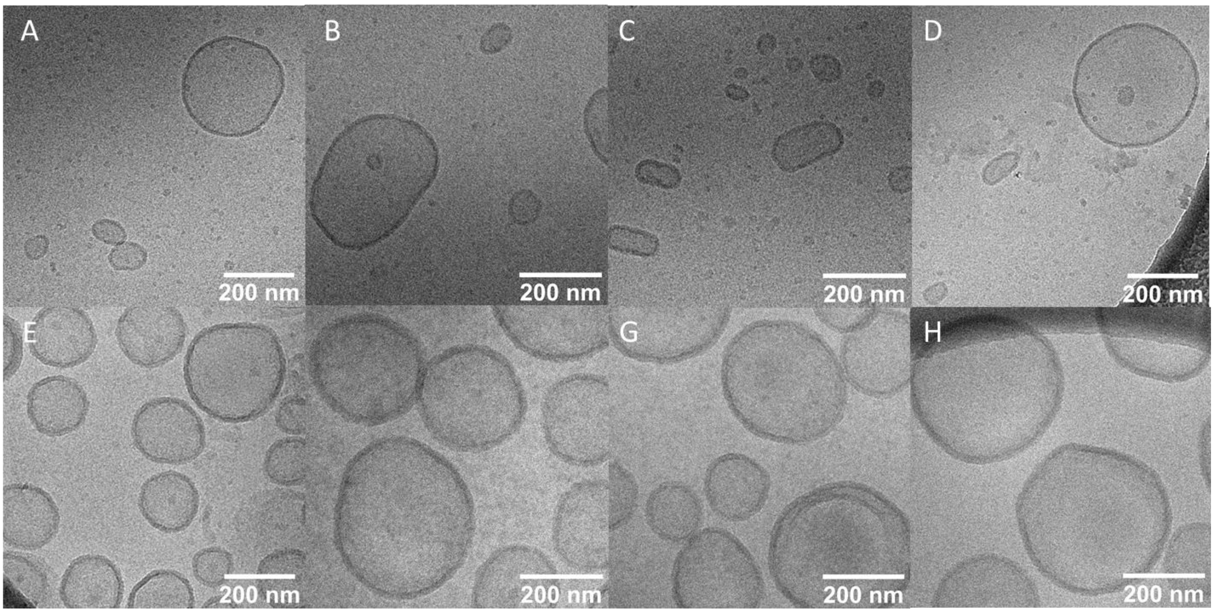

Supplementary Figure 6 │cryo-TEM micrographs of the synthesised nanoreactors. A-D. DASA-esterase. **E-H**. PISA-urease.

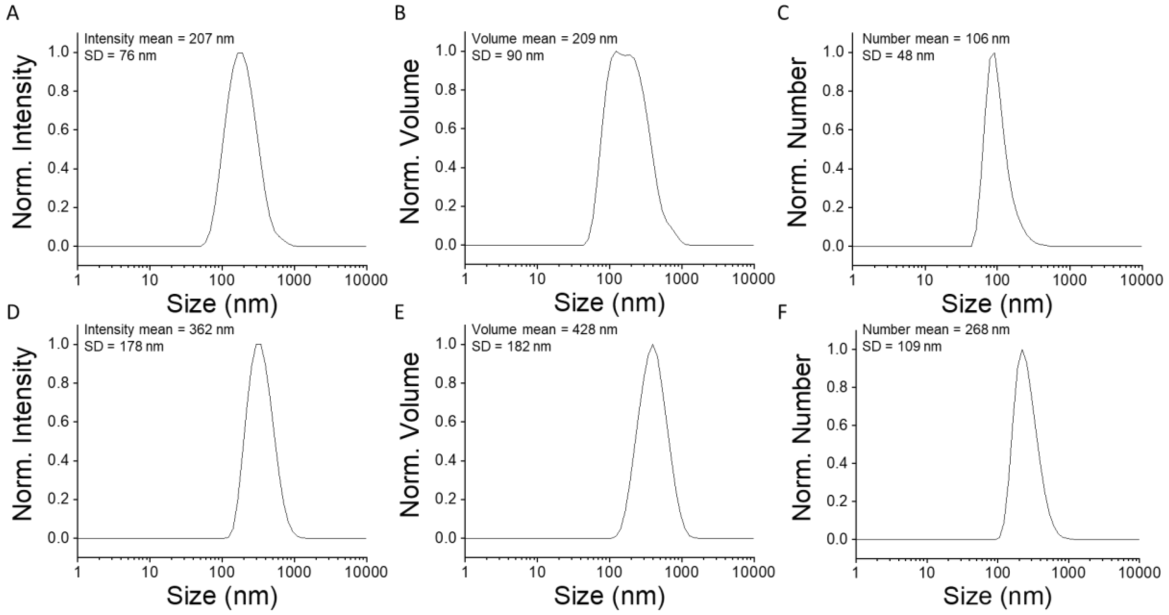

Supplementary Figure 7 │DLS of the synthesised nanoreactors. A-C. DASA-esterase. **D-F**. PISA-urease. Data shown as mean (average size of 3 repeated measurements).

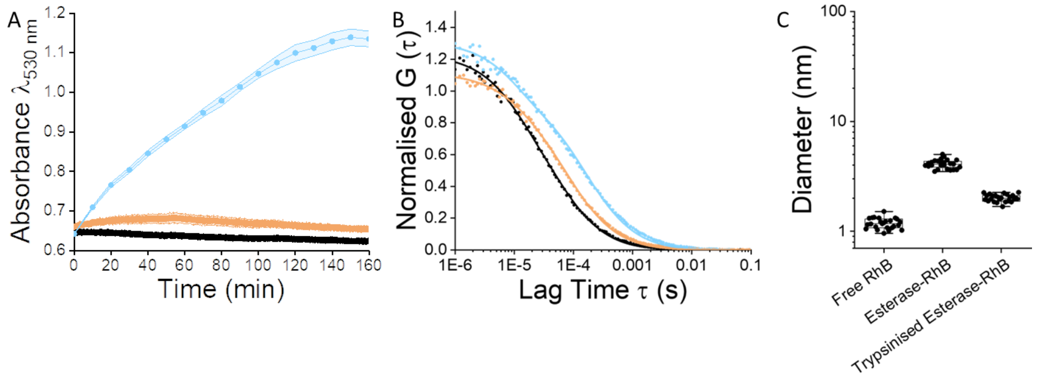

Supplementary Figure 8 │Digestion of non-encapsulated esterase with trypsin. A. UV-Vis kinetics of ethyl acetate hydrolysis reaction catalysed by free trypsinised esterase (orange), DASA-esterase nanoreactors with externally trypsinised esterase employing green light (λ = 530 nm) at 1.49 mW·cm^{-2} (blue) and in darkness (black) (*n* = 3 technical replicates, mean ± SD). The last two datasets were reproduced from Figure 2D for illustration purposes. **B**. Normalised FCS autocorrelation curves of RhB-esterase (blue), trypsinised RhB-esterase (orange), and sulforhodamine B (black) (average curves of *n* = 25 technical replicates, 5 s each, dots represent raw data, fitted curves are straight lines). **C**. Hydrodynamic diameters derived from Normalised FCS autocorrelation curves in panel B (displayed: centre line, the median; box limits, upper and lower quartiles; whiskers, minimum and maximum values, *n* = 25 technical replicates, 5 s each).

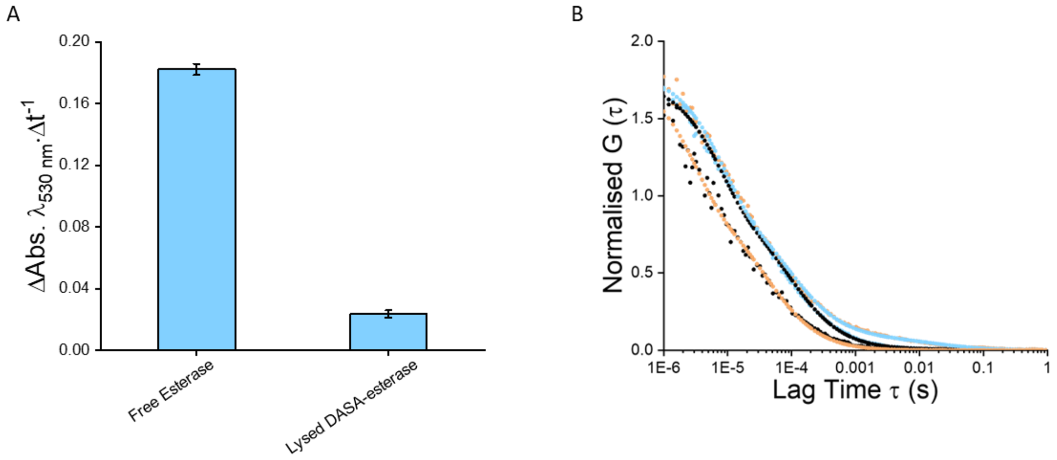

Supplementary Figure 9│Encapsulation of esterase in DASA-derived polymersomes. **A**. Hydrolysis of ethyl acetate in the presence of MR catalysed by free esterase and lysed DASA-esterase nanoreactors. The absorbance values were plotted after 3 min (*n* = 3 technical replicates, mean ± SD). **B**. Normalised FCS autocorrelation curves of DASA-esterase-AF647 (orange), esterase-AF647 with spiked empty DASA polymersomes (blue), and Alexa Fluor 647 (black) (average curves of n = 25 technical replicates, 5 s each, dots represent raw data, fitted two-component curves are straight lines).

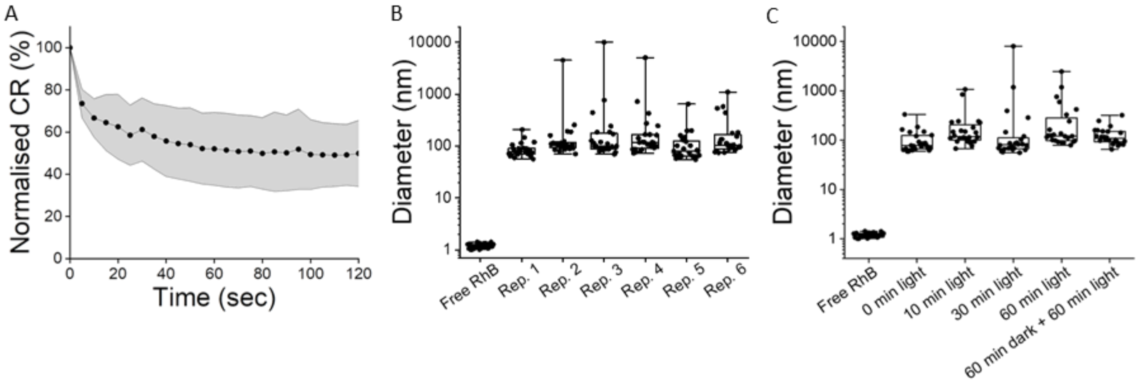

Supplementary Figure 10 │Single particle characterisation of DASA photoswitching. DASAs isomerise from a colourful, fluorescent, and less polar isomer in darkness to a colourless, non-fluorescent, and more polar isomer in the presence of visible light. **A**. FCS normalised count rate (CR) of DASA polymersomes excited with a laser (λ_{ex} = 561 nm) over time (6 samples with *n* = 25 technical replicates, 5 s each, mean ± SD). **B**. Hydrodynamic diameters derived from FCS autocorrelation analysis of raw CR panel B. **C**. Hydrodynamic diameters derived from normalised FCS autocorrelation curves of samples externally irradiated with green light (λ_{ex} = 530 nm) (*n* = 25 technical replicates, 5 s each; displayed: centre line, the median; box limits, upper and lower quartiles; whiskers, minimum and maximum values).

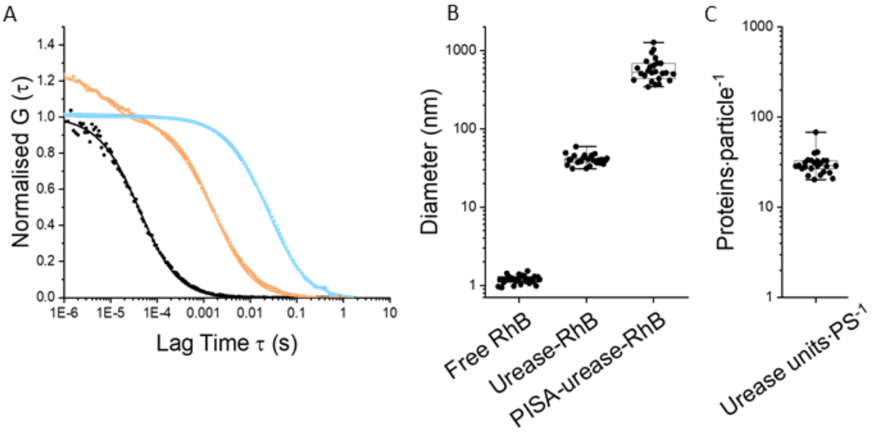

Supplementary Figure 11 │FCS measurements showing the encapsulation of urease in PISA-derived polymersomes. **A.** Normalised FCS autocorrelation curves of PISA-RhB-urease (blue), RhB-urease (orange), and sulforhodamine B (black) (average curves of *n* = 25 technical replicates, 5 s each, dots represent raw data, fitted curves are straight lines). **B.** Hydrodynamic diameters derived from normalised FCS autocorrelation curves in panel A **C.** Quantification of urease proteins per PISA polymersome. (Displayed: centre line, the median; box limits, upper and lower quartiles; whiskers, minimum and maximum values, *n* = 25).

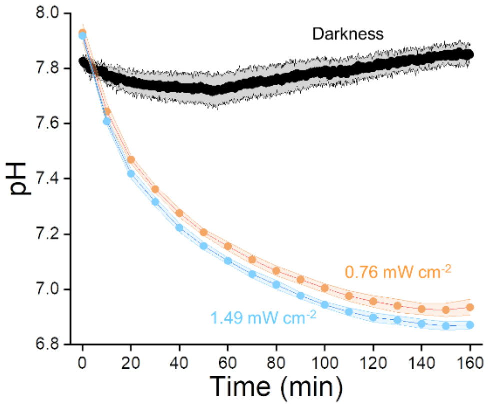

Supplementary Figure 12 | DASA-esterase nanoreactor mediated biocatalytic hydrolysis of ethyl acetate in the presence of MR. Evolution of pH by irradiation of green light (λ = 530 nm) at 1.49 mW·cm^{-2} (blue), 0.76 mW·cm^{-2} (orange), and darkness (black). The formation of MRH was monitored by absorbance measurements at 530 nm (*n* = 3 technical replicates, mean ± SD) shown in Figure 2D in the main manuscript.

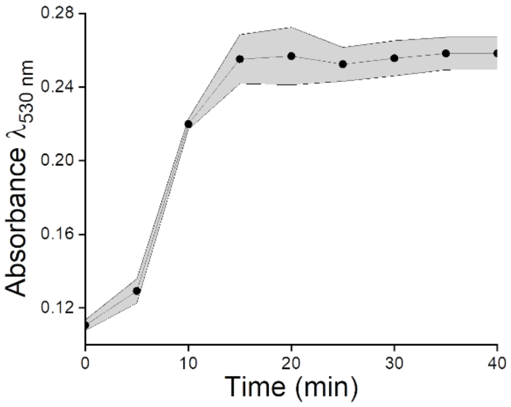

Supplementary Figure 13 | Production of MRH from MR$^-$ at low concentration. UV-Vis kinetic measurements catalysed by DASA-esterase under continuous irradiation of green light (λ = 530 nm) (black) (*n* = 3 technical replicates, mean ± SD). The plateau absorbance values corresponded with the absorbance of MR at pH = 1.8.

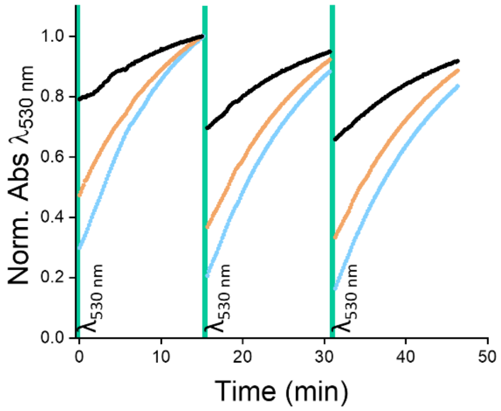

Supplementary Figure 14 |UV—Vis measurements of DASA polymer photoswitching in THF. In each photoswitching cycle, the solutions were irradiated for 30 sec and thermal recovery of the absorbance was monitored at 530 nm for 15 min. The samples were irradiated at green light (λ = 530 nm) intensities of 0.23 mW·cm^{-2} (black), 0.76 mW·cm^{-2} (orange), and 1.49 mW·cm^{-2} (blue).

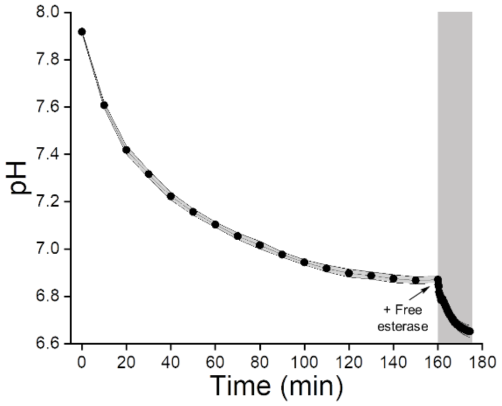

Supplementary Figure 15 | Evolution of pH after addition of free esterase after formation of green light-mediated plateau. 60 pmol of free esterase was added after the formation of plateau by green light at 1.49 mW·cm^{-2} (n = 3 technical replicates, mean ± SD). The data was calculated from the absorbance values shown in Figure 2F in the main manuscript.

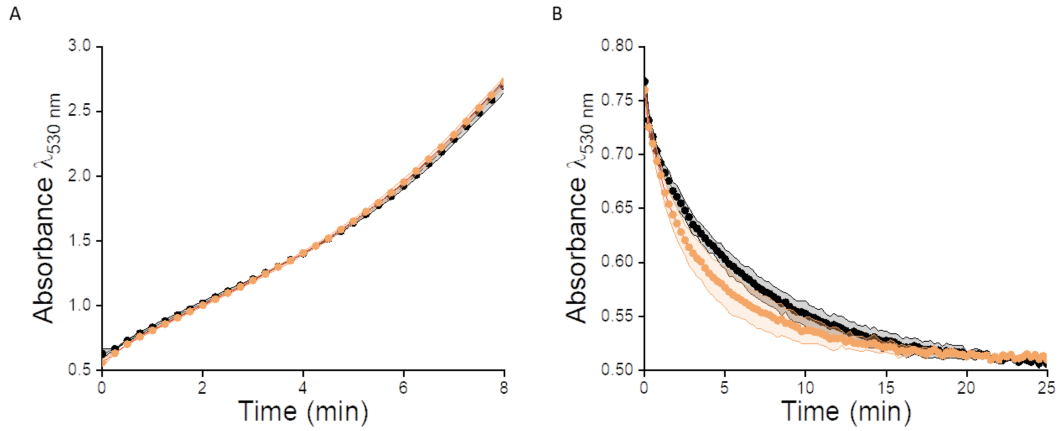

Supplementary Figure 16 |Stability of esterase and urease to light at 1.49 mW·cm^{-2}. UV-Vis kinetic measurements of MR protonation state**. A.** Esterase catalysed hydrolysis of ethyl acetate in the presence of MR^{-} after pre-treatment of esterase with either green light (λ = 530 nm) for 160 min (orange) or darkness (black) (n = 3, technical replicates, mean ± SD). **B**. Urease catalysed hydrolysis of urea in the presence of MRH after pre-treatment of urease with either green light (λ = 530 nm) for 160 min (orange) or darkness (black) (n = 3, technical replicates, mean ± SD).

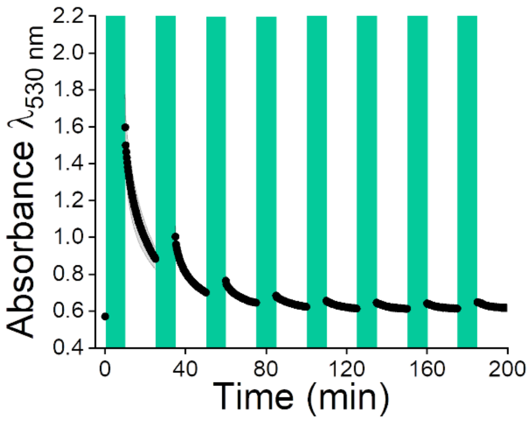

Supplementary Figure 17 | Modulation of medium species by alternating light between DASA-esterase and PISA-urease nanoreactors. UV-Vis measurements monitoring the formation of MRH by alternation of green light at 4.31 mW·cm^{-2} and darkness. In each cycle, the samples were irradiated for 10 min and the absorbance at λ = 530 nm was probed for 15 min in darkness (*n* = 3, technical replicates, mean ± SD). The pH evolution in Figure 4B in the main manuscript was calculated from such absorbance values.

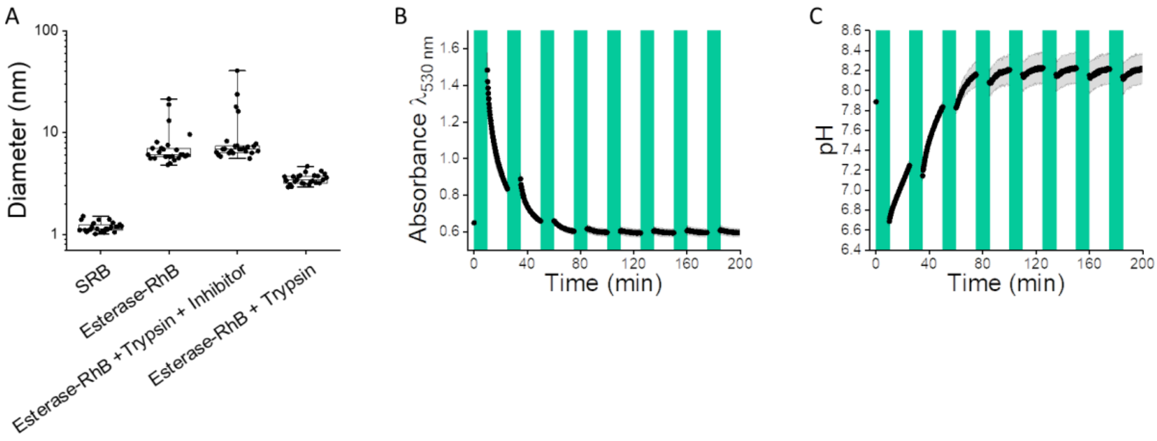

Supplementary Figure 18 | Light-mediated modulation of pH by chemical communication between DASA-esterase and PISA-urease nanoreactors in the presence of a trypsin inhibitor. A. FCS-derived hydrodynamic diameters of esterase-RhB in the presence of trypsin alone and in the presence of trypsin that was first pre-incubated with the trypsin inhibitor, aprotinin (*n* = 25 technical replicates, 5 s each; displayed: centre line, the median; box limits, upper and lower quartiles; whiskers, minimum and maximum values). **B**. UV-Vis measurements monitoring the formation of MRH by alternation of green light (λ = 530 nm) at 4.31 mW·cm^{-2} and darkness in the presence of aprotinin. In each cycle, the samples were irradiated for 10 min and the absorbance at λ = 530 nm was probed for 15 min in darkness (*n* = 3, technical replicates, mean ± SD). **C**. pH evolution calculated from absorbance values in panel B.

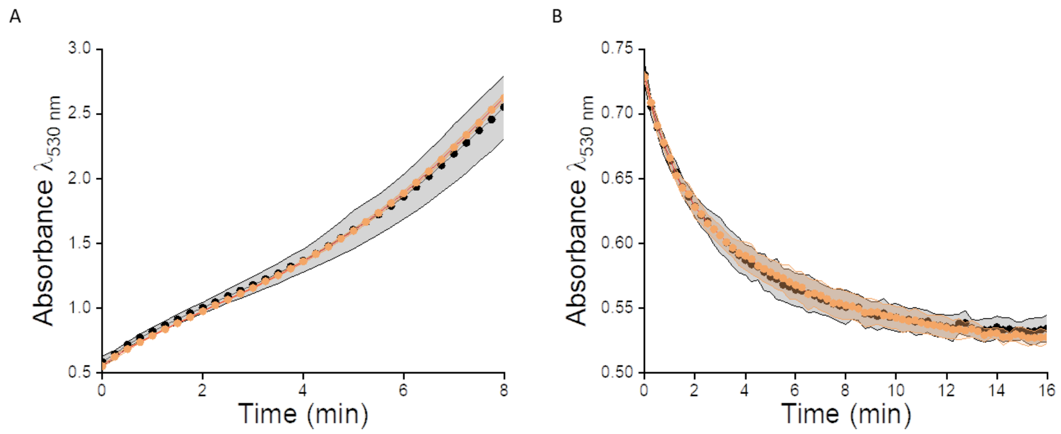

Supplementary Figure 19 │Stability of esterase and urease to light at 4.31 mW·cm^{-2}. UV-Vis kinetic measurements of MR protonation state**. A.** Esterase catalysed hydrolysis of ethyl acetate in the presence of MR$^-$ after pre-treatment of esterase with either green light (λ = 530 nm) for 80 min (orange) or darkness (black) (*n* = 3, technical replicates, mean ± SD). **B**. Urease catalysed hydrolysis of urea in the presence of MR$^-$ after pre-treatment of urease with either green light (λ = 530 nm) for 80 min (orange) or darkness (black) (*n* = 3, technical replicates, mean ± SD).

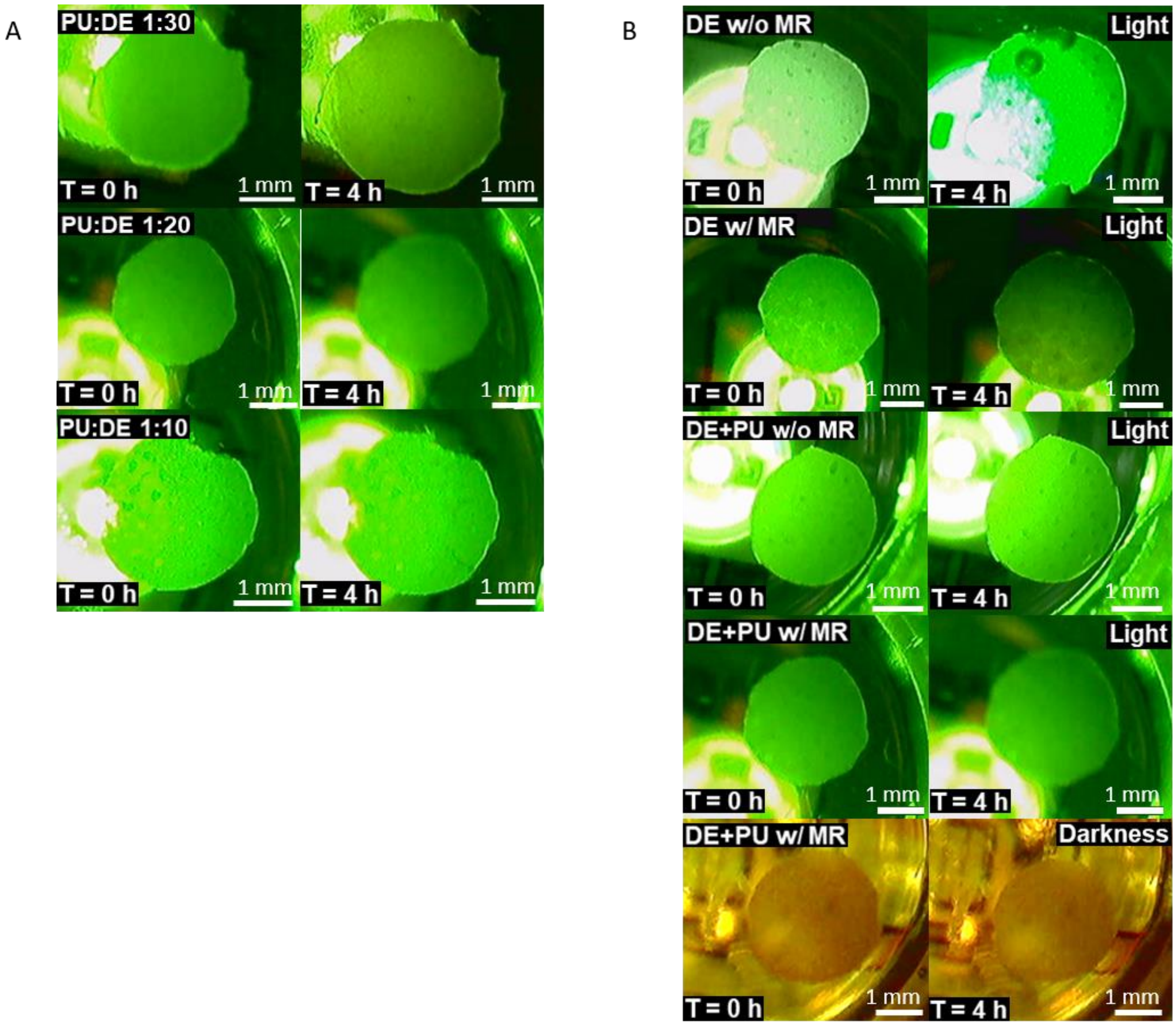

Supplementary Figure 20 | Modulation of hydrogel swelling by communication between antagonistic nanoreactors. Photographs of hydrogel swelling states performed in 96-well plates. The images were normalised to the distance corresponding to the bottom of the wells (9 mm). The scale bar corresponds to 1 mm. **A**. The images correspond to the measurements shown in Figure 5B at ratios of PU:DE 1:30 (top), 1:20 (middle), and 1:10 (bottom) throughout irradiation with green light (λ = 530 nm) at 4.31 mW cm^{-2} from t = 0h (left) to t = 4h (right). **B**. The images correspond to the measurements shown in Figure 5C catalysed by DASA-esterase in absence of MR (top), DASA-esterase in the presence of MR (second), DASA-esterase and PISA-urease in absence of MR (third), DASA-esterase and PISA-urease in the presence of MR (fourth), and DASA-esterase and PISA-urease in darkness from t = 0 h (left) to t = 4 h (right).

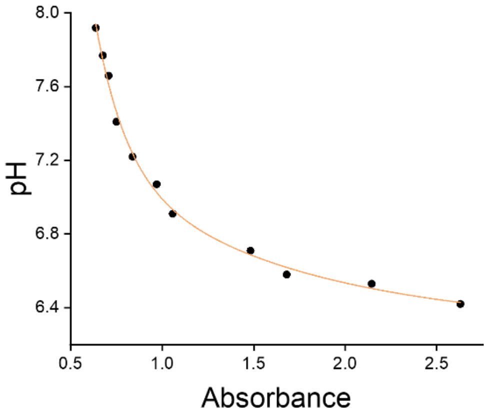

Supplementary Figure 21 │Calibration curve of absorbance vs pH. The aqueous solutions were composed of MR ($7.4 \cdot 10^{-1}$ µmol·mL^{-1}), urea ($8.3 \cdot 10^{-1}$ mmol·mL^{-1}), and saturated ethyl acetate at a range of pH and absorbance values. The data was fitted to an exponential decay function ($r^2 = 0.99$).

Supplementary References

1. Wrobel, M. M.; Bánsági Jr, T.; Scott, S. K.; Taylor, A. F.; Bounds, C. O.; Carranza, A.; Pojman, J. A., pH wave-front propagation in the urea-urease reaction. *Biophys. J.* **2012,** *103* (3), 610-615.
2. Adler, A. J.; Kistiakowsky, G., Kinetics of pig liver esterase catalysis. *J. Am. Chem. Soc.* **1962,** *84* (5), 695-703.
3. Cheng, Z.; Tsourkas, A., Paramagnetic porous polymersomes. *Langmuir* **2008,** *24* (15), 8169-8173.
4. Che, H.; Cao, S.; van Hest, J. C., Feedback-induced temporal control of "breathing" polymersomes to create self-adaptive nanoreactors. *J. Am. Chem. Soc.* **2018,** *140* (16), 5356-5359.
5. Wang, X.; Moreno, S.; Boye, S.; Wen, P.; Zhang, K.; Formanek, P.; Lederer, A.; Voit, B.; Appelhans, D., Feedback-Induced and Oscillating pH Regulation of a Binary Enzyme–Polymersomes System. *Chem. Mater.* **2021**.
6. Wang, X.; Hu, J.; Liu, G.; Tian, J.; Wang, H.; Gong, M.; Liu, S., Reversibly switching bilayer permeability and release modules of photochromic polymersomes stabilized by cooperative noncovalent interactions. *J. Am. Chem. Soc.* **2015,** *137* (48), 15262-15275.
7. Rifaie-Graham, O.; Ulrich, S.; Galensowske, N. F.; Balog, S.; Chami, M.; Rentsch, D.; Hemmer, J. R.; Read de Alaniz, J.; Boesel, L. F.; Bruns, N., Wavelength-Selective Light-Responsive DASA-Functionalized Polymersome Nanoreactors. *J. Am. Chem. Soc.* **2018,** *140*, 8027−8036.
